# Responsible remembering: The role of metacognition, forgetting, attention, and retrieval in adaptive memory

**DOI:** 10.3758/s13423-024-02554-9

**Published:** 2024-08-13

**Authors:** Dillon H. Murphy

**Affiliations:** 1 University of California Los Angeles, Los Angeles, CA USA; 2 Department of Psychology, Arizona State University, Tempe, AZ USA; 3 University of California, Riverside, CA USA

**Keywords:** Responsible remembering, Metamemory, Forgetting, Retrieval

## Abstract

In our everyday lives, we must remember important information, especially if there are consequences for forgetting. In this review, I discuss recent work on *responsible remembering*: the strategic and effortful prioritization of important information with consequences for forgetting. Thus far, research regarding responsible remembering has revealed several key factors and mechanisms that work together to enhance memory for important information that will continue to be refined: the identification and selection of what to remember (metacognitive reflectivity), the forgetting of less important information to facilitate memory for items that do need to be remembered (responsible forgetting), the functional prioritization of attention at the expense of competing factors (responsible attention), and the selective recall of important information via efficient retrieval strategies (responsible retrieval). Together, these functions form a cohesive system that aims to selectively prioritize, encode, and recall information that is deemed important based on its anticipated utility or the consequences of forgetting, and considering the importance of information may be a critical memory adaptation as we age. Specifically, if younger and older adults learn to self-assess and prioritize important information that has negative consequences if forgotten, engage in strategic forgetting, efficiently allocate their attentional resources, and utilize effective retrieval operations, memory for said important information can be enhanced.

Whether remembering items on a shopping list, children’s allergies, or items to pack for a vacation, we are often exposed to more information than can be remembered. When attempting to retain large amounts of information, people should strategically focus on important information to maximize the likelihood that this information will be effectively encoded and later remembered. For example, if people fail to remember important information, the consequences for forgetting could have disastrous repercussions, such as giving a child food containing a known allergen or forgetting to pack your passport for a vacation getaway. Examining people’s understanding of how their memory works (i.e., metamemory), what information they try to remember and forget, as well as the underlying attentional processes, retrieval operations, and adaptive mechanisms that contribute to memory for important information can help broaden our understanding of how and why people achieve memory for important information and avoid consequences for forgetting.

In this paper, I introduce a new theoretical framework—*responsible remembering*—that presents remembering as a process uniquely tailored to the importance of the material. This approach posits that memory is not merely a passive store of information but an adaptive system that prioritizes information based on its perceived value. The responsible remembering framework seeks to explain how and why certain memories are preserved over others, suggesting that metacognition, attention, encoding, and retrieval processes interact to enhance memory for the most consequential information for an individual’s needs and goals.

By examining these mechanisms in concert, I propose a model (see Fig. 1) that elucidates the complex interplay between the cognitive processes involved in responsible remembering. Specifically, responsible remembering begins with the intake of incoming information, which triggers metacognitive reflectivity. Here, individuals assess the importance of the information and the consequences of forgetting it. This evaluation leads to a bifurcation where important information, recognized for its potential consequences if forgotten, is subjected to responsible attention. This involves the attentional resources required for selective studying and encoding to ensure important information is effectively encoded in memory while less critical information may be strategically forgotten to reduce cognitive load. This selective attention then feeds into responsible retrieval where strategic retrieval processes are employed to recall important information effectively. The culmination of these processes results in responsible remembering where critical information is recalled successfully and less important information is efficiently forgotten. This responsible remembering model emphasizes the impact of each step in ensuring that memory serves the adaptive function of prioritizing information based on its importance.

**Fig. 1:**
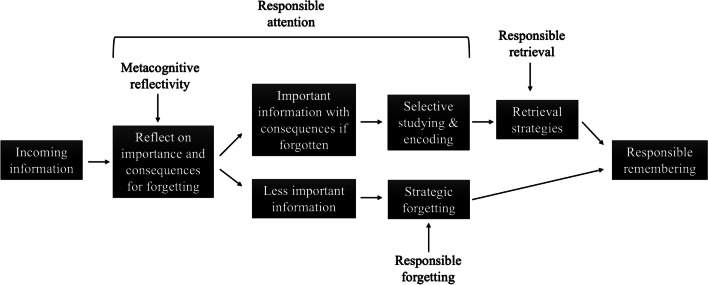
How metacognitive reflectivity, responsible forgetting, responsible attention, and responsible retrieval contribute to responsible remembering

The conceptualization of responsibly remembering information according to its “importance” can be considered in two distinct yet interrelated ways. First, responsible remembering pertains to the task-specific importance assigned to information, such as when experimenters attribute arbitrary values to to-be-remembered items. Here, the importance is defined externally by the conditions of the task, guiding participants to prioritize certain information based on predetermined values. Second, and perhaps more importantly, responsible remembering addresses the inherent, universal needs of our memory system to prioritize and retain information that is crucial for our well-being and daily functioning. This form of remembering is driven by an intrinsic understanding of the utility and potential consequences associated with retaining or forgetting specific pieces of information (similar to need probability, see Anderson & Schooler, 1991, 2000; Anderson et al., 1997). For instance, remembering to take critical medication has obvious health benefits, while forgetting to do so can have serious health repercussions. This broader understanding of information’s importance emphasizes memory’s adaptive role in helping us navigate complex environments, ensuring we remember what is most beneficial for our survival and success, and avoid the negative outcomes that come from forgetting vital information.

While many fundamental learning and memory processes are shared across species, such as rats and pigeons, the concept of responsible remembering involves a more complex interaction between conscious and strategic processes that anticipate future needs which may be unique to human cognition. This type of memory processing involves not only recognizing the intrinsic importance of information but also actively managing memory resources to enhance the retention of this information. While incidental learning can be effective and often parallels intentional learning in overall performance—provided it engages deep processing—incidental learning typically does not inherently prioritize information based on its importance. Responsible remembering, therefore, goes beyond the mere acquisition of information; it involves a deliberate effort to select, encode, and retrieve information deemed important based on perceived future utility rather than mere exposure, and these interrelated mechanisms likely work together to achieve responsible remembering.

## Value-directed remembering and metacognition

To examine memory for valuable information in the lab, Castel et al. (2002) presented participants with words paired with point values that count toward participants’ scores if recalled. With their goal being to maximize their point scores, participants tend to optimize task performance by best recalling the most valuable information, often at the expense of low-value items (Ariel et al., 2009; Castel, 2008a; Castel et al., 2007, 2013; Elliott, McClure et al., 2020b; Murphy, 2023a; Soderstrom & McCabe, 2011; see Knowlton & Castel, 2022; Madan, 2017, for review). Thus, people can use value to guide memory, but the evaluation and monitoring of one’s memory processes may also play a role.

In a seminal study, McGillivray and Castel (2011) used a similar value-directed remembering paradigm but required participants to “bet” on whether they would later recall each word. If participants bet on and later recalled a word, they received the points associated with that word. However, if they bet on a word and failed to recall it, they lost the points associated with that word. If participants did not bet on a word, points were neither gained nor lost regardless of the learning outcome. Results revealed that participants (particularly older adults) demonstrated strategic betting behavior and selectively remembered high-value words, particularly after gaining task experience. Thus, this simulated a situation with rewards for remembering and costs for forgetting demonstrated that metacognition and learning outcomes can be enhanced when learners are faced with consequences for forgetting.

Asking participants to make predictions about memory for presented words, such as betting on whether a word will be remembered, requires an awareness and understanding of one’s memory processes (i.e., metamemory; Dunlosky et al., 2016; Nelson, 1996). Metamemory refers to an individual’s knowledge and awareness of their own memory capabilities and processes. This self-awareness plays a critical role in how effectively one can monitor and regulate memory function, influencing decisions about which strategies to employ for encoding, storing, and retrieving information. Specifically, in Nelson and Narens’s (1990) framework of metamemory, they differentiate two major processes: metacognitive monitoring and control. Measures of monitoring typically involve evaluating the likelihood of remembering something and, rather than a binary “betting” prediction, often take the form of judgments of learning (JOLs): metacognitive self-assessments of how likely one is to remember information on a later test (see Rhodes, 2016). Most measures of monitoring, such as JOLs^1^, are assessed as a probability, or percentage likelihood (on the same scale as the probability of recall), allowing for the examination of the *absolute* and *relative accuracy* of participants’ judgments.

Absolute accuracy, referred to as *calibration*, is the overall relationship between judgments and performance and is measured by the difference between participants’ average judgments and the percentage of information recalled. For example, if a participant’s average JOL exceeds performance, this would indicate overconfidence, but if performance surpasses a participant’s average JOL, this indicates underconfidence. Good calibration is indicated by a close correspondence between JOL magnitude and memory performance. However, a well-calibrated participant (i.e., someone correctly estimating their overall memory ability) may not be aware of the specific items that will be remembered or forgotten.

*Relative accuracy*, referred to as *resolution*, is the degree to which an individual remembers the specific information they indicated that they would remember (see Higham et al., 2016; Rhodes, 2016) and is often measured by Gamma correlations (Gonzalez & Nelson, 1996; Nelson, 1984) or multilevel models with memory accuracy predicted by JOLs (Murayama et al., 2014; Vuorre & Bolger, 2018; see Masson & Rotello, 2009, for alternative approaches). An individual with good resolution gives higher JOLs for information that is later remembered and lower JOLs for information that is later forgotten. Thus, good relative accuracy exemplifies the ability to distinguish between what will or will not be remembered; the individual is neither overconfident nor underconfident and remembers the information that they predict that they will remember.

Although JOLs are often related to the difficulty of initial learning (Hertzog & Dunlosky, 2011) and can involve the effortful and strategic incorporation of multiple cues (Undorf & Bröder, 2019), JOLs are sometimes based on factors that have little impact on memory performance (see Rhodes, 2016; Schwartz & Efklides, 2012, for factors that affect JOL accuracy). Specifically, to maximize accuracy, JOLs should incorporate the cues that impact memory performance and ignore those that have minimal effects. However, participants often incorporate cues that have little effect on memory performance, resulting in a weak relationship between judgments and performance in these instances (e.g., Besken & Mulligan, 2013; Kornell et al., 2011; Mueller & Dunlosky, 2016; Rhodes & Castel, 2009). For example, Rhodes and Castel (2008) presented participants with to-be-remembered words in either large or small font and found that participants rated words presented in large font as more likely to be remembered than words in a small font, but font size did not significantly affect recall (but see Chang & Brainerd, 2022; Luna et al., 2018; see Ball et al., 2014, for a similar demonstration using educationally relevant materials).

While the influence of processing fluency (e.g., font size) on JOLs can lead to a weaker relationship between metamemory and later remembering, JOLs are generally sensitive to an item’s value. For example, Murphy et al. (2021) found that participants rated high-value words as more likely to be remembered and low-value words as less likely to be remembered, demonstrating a metacognitive awareness of value-directed remembering. Furthermore, this sensitivity of participants’ JOLs to item value was associated with greater selective memory for high-value words and increased as the task endured. Additionally, participants’ metacognitive awareness of selectivity (i.e., the positive relationship between JOLs and item value) and metacognitive accuracy also increased with task experience, indicating that participants become more aware of their limited memory capacity and are more strategically selective with what they remember with increased task experience.

**Fig. 2:**
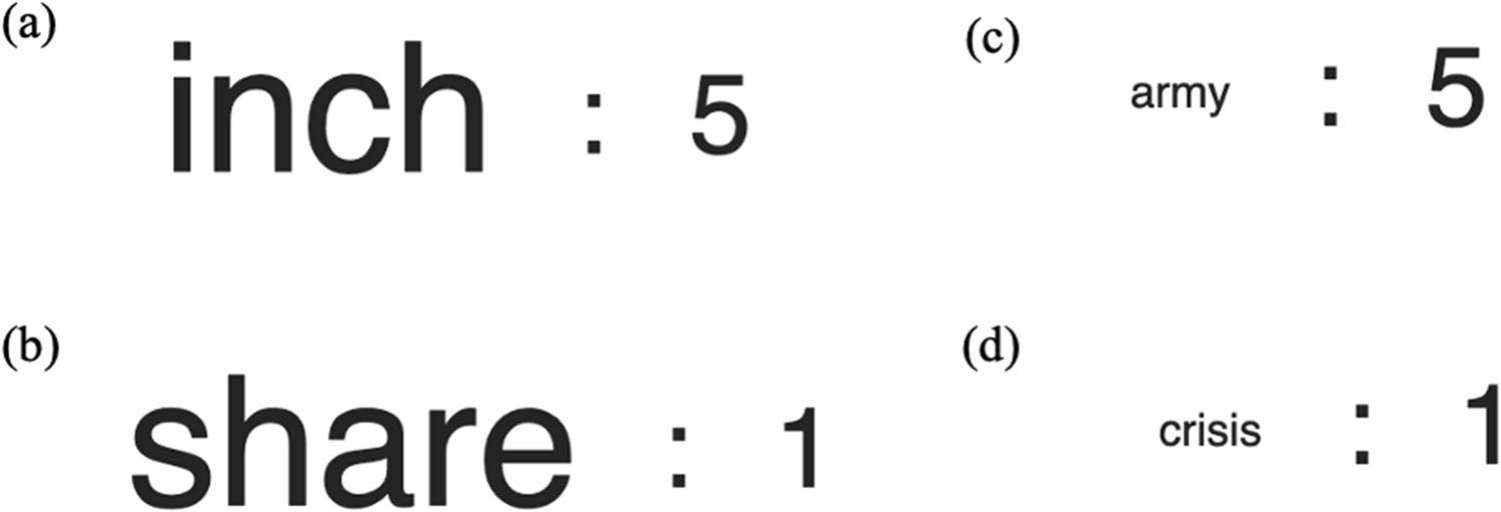
Example study phase with large font and high-value (**a**), large font and low-value (**b**), small font and high-value (**c**), and small font and small-value (**d**) in Murphy, Huckins et al. 2022a, Murphy, Schwartz et al. 2022b). Participants were more sensitive to value than font size in both their judgments and recall

Although both perceptual processing fluency and value can influence participants’ JOLs (see also Soderstrom & McCabe, 2011), fluency and value differentially impact recall and metacognitive judgments. For example, Murphy, Huckins et al. (2022a) demonstrated that when words in large and small fonts are paired with high or low values (see Fig. 2), both participants’ JOLs and recall are more sensitive to an item’s point value than its font size. Specifically, high-value words were better remembered than low-value words, regardless of font size, and participants were generally metacognitively aware of their selectivity for valuable information. Thus, certain cues available at encoding are more influential in both memory and metacognition than others.

People may be more likely to predict greater memorability for words in a larger font than those in a smaller font due to their inaccurate beliefs about the factors that impact memory (Mueller et al., 2014; Undorf & Zimdahl, 2019). For example, Luna et al. (2019) explored the impact of font size on memory—specifically, how larger fonts can lead to higher JOLs due to perceived importance. They presented participants with words in different font sizes and assessed participants’ JOLs, judgments of importance, and actual memory performance. Results revealed that words in larger fonts were not only perceived as more important but also remembered better. This effect was not attributable to increased processing fluency such that larger fonts decreased processing speed. These results suggest that the relationship between font size and memory performance is mediated by the perceived importance of the information, rather than by perceptual ease or fluency. Thus, learners might consider font size as a crucial predictor of future memorability and incorporate it into their metamemory judgments. Additionally, learners may associate larger font size with importance, as is the case with headlines compared with footnotes, and use this assessment of importance to guide memory (Murphy & Castel, 2021a, 2021b; Murphy, Hoover et al., 2023c). Therefore, using font size as a memory guide might be a beneficial strategy to increase retention if learners believe that information in a larger font is more important than that in a smaller font.

To further examine the role of beliefs about processing fluency and value in memory, Murphy, Rhodes et al. (2024e) presented participants with words in varying font sizes, where larger fonts were associated with either higher or lower point values that counted towards participants’ scores if they could recall them later. However, the point values associated with each word were not explicitly stated to participants—they had to figure out the relationship between font size and value on their own (via feedback at the end of each list). When larger fonts were linked with higher point values, participants showed better recall of high-value words compared with low-value words, but participants were not selective with their memory when smaller fonts were linked with higher point values. Therefore, if the value or importance of information aligns with the learners’ beliefs, they may be more capable of selectively remembering valuable information, and learners’ metacognitive judgments should incorporate their beliefs.

Many metacognitive measures, like JOLs, occur during the encoding phase such that judgments are made immediately after an item is studied (rather than before encoding, see Castel, 2008b; or retrospective judgments occurring after a memory test, see Busey et al., 2000; Dougherty et al., 2005). As a result, these monitoring assessments are often informed by the cues available during learning. Koriat (1997) proposed a cue-utilization framework in which three types of cues inform monitoring assessments: *intrinsic cues* (characteristics of items that influence or are believed to influence memory such as word-pair relatedness)*, extrinsic cues* (the encoding operations used by the learner as well as the conditions of encoding and testing like presentation rate or testing recall versus recognition)*, *and *mnemonic cues *(past experience with items such as how easily an item comes to the learner’s mind in response to a cue). This framework has been frequently supported (e.g., Broder & Undorf, 2019; Koriat, 2015) and generally leads to accurate predictions when judgments and performance are based on these same factors (e.g., item relatedness; see Dunlosky & Matvey, 2001; Rhodes & Tauber, 2011; Tiede & Leboe, 2009).

Using cues with poor predictive validity of later remembering (i.e., using font size rather than value) could lead to inefficient encoding strategies (such as a poor allocation of study time, see Metcalfe & Finn, 2008) and subsequently poorer memory outcomes. While JOLs serve as a measure of metacognitive monitoring, the self-regulation of study time and study choices is a metacognitive control process based on information gained from monitoring (Dunlosky et al., 2016; Egner, 2017; Nelson, 1996; Nelson & Narens, 1990; Son & Metcalfe, 2000; Thiede & Dunlosky, 1999; but see Koriat et al., 2006, for the control affects monitoring perspective). One useful theoretical framework of these control processes, agenda-based regulation (Ariel, 2013; Ariel & Dunlosky, 2013; Ariel et al., 2009; Dunlosky & Ariel, 2011a, 2011b), posits that when presented with to-be-remembered information, learners develop and use goal-oriented agendas based on monitoring assessments to inform control processes and focus on what they need to know. Thus, the interplay between metacognitive monitoring and control can be crucial for the strategic allocation of attention and the subsequent remembering of important information.

### Responsible remembering

Accurately predicting recall exemplifies good metacognition, but people often have difficulty anticipating future forgetting (Koriat et al., 2004; Kornell et al., 2011). For example, most people have had experiences where they expected to remember important information, like names or anniversaries, but forgot it at an inopportune time and had to deal with the consequences like social embarrassment or an angry spouse. In some extreme cases, the consequences for unexpected forgetting can be deadly, such as distracted parents forgetting infants in the back seats of hot parked cars (see Anselmi et al., 2020)—something that seems unimaginable to those who have not experienced it.

Minor instances of forgetting can be inconvenient and failing to consider the potential (although sometimes minimal) consequences of forgetting can contribute to instances of inaccurate metacognition (e.g., Serra & England, 2012). However, since the most important information is usually that which has the most severe outcomes if forgotten, situations involving negative consequences for forgetting often lead to improved metacognition and learning outcomes (e.g., McGillivray & Castel, 2011). How our memory adaptively functions to prioritize important information that will need to be remembered as well as how metacognitive processes may be more precise in situations involving consequences for forgetting is a notion termed *responsible remembering* (Murphy & Castel, 2020).

**Fig. 3:**
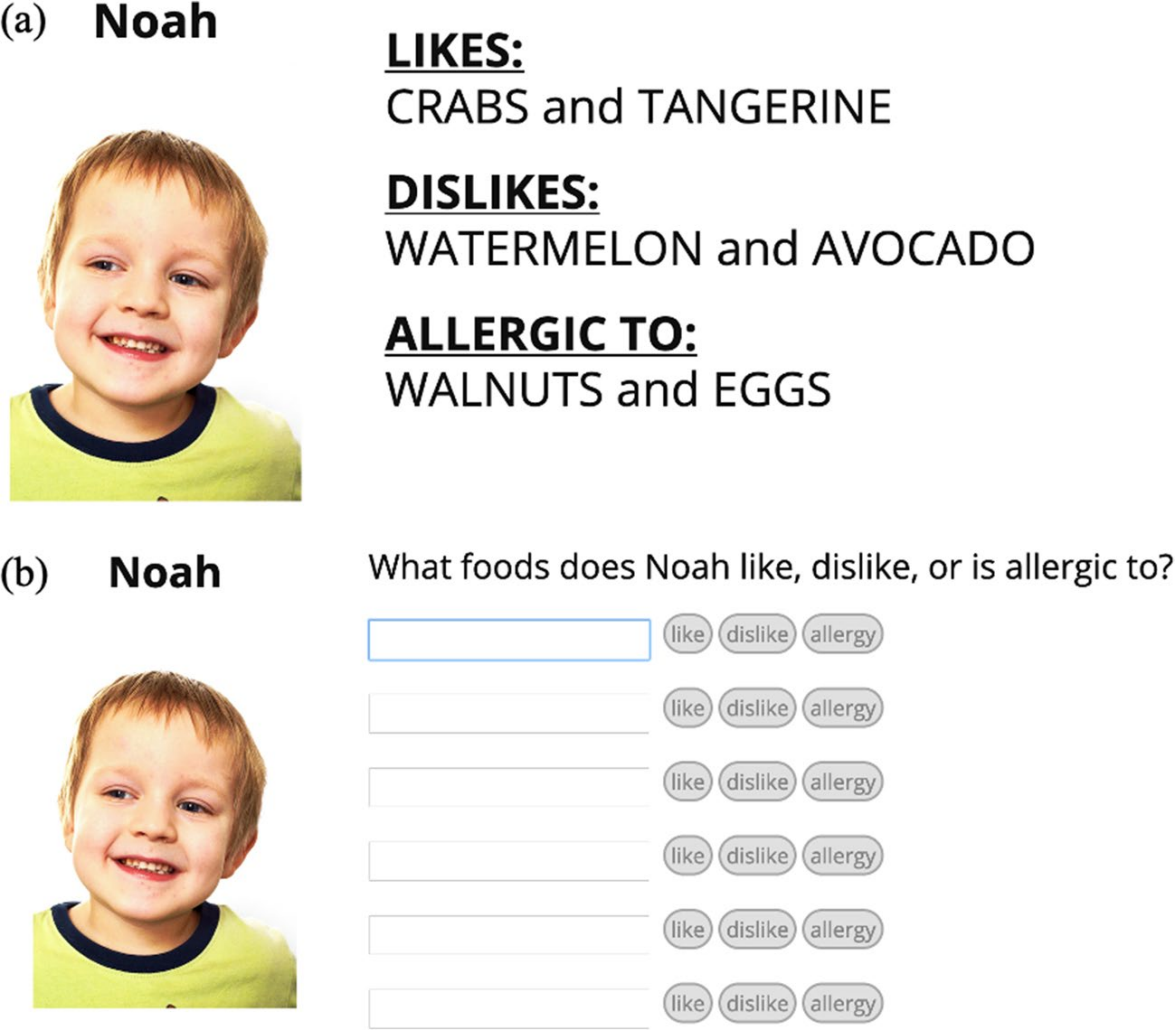
Example of the study phase (**a**) and test phase (**b**) in Murphy and Castel (2021a) and Murphy, Hoover et al., (2023c)

*Responsible remembering*, a form of adaptive memory (see Nairne, 2010, 2013, 2015; Nairne & Pandeirada, 2008, 2010; Nairne et al., 2007), captures one’s knowledge of selective memory processes and allows for the efficient use of memory in a variety of contexts. To demonstrate a situation where participants engage in responsible remembering, Murphy and Castel (2021a) presented participants with lists of children and their associated food preferences to remember for a later test (foods the children like, dislike, or are allergic to and must avoid, see Fig. 3). When participants were forced to consider the importance of remembering each child’s food preferences (rather than passively monitoring their learning), information with consequences for forgetting (the children’s allergies) was deemed most important and subsequently best remembered.

In a follow-up study, Murphy, Hoover, et al. (2023c) used a similar paradigm but allowed participants to self-regulate their study of the children’s food preferences. Results revealed that when participants were asked to consider the importance of remembering each child’s food preferences (by making a judgment of importance for each category of items), they subsequently spent more time studying the children’s allergies, revealing one potential mechanism of their superior memory for the information with consequences if forgotten. Specifically, participants’ reflection on the importance of remembering information resulted in a strategic allocation of study time towards this information to minimize the potential consequences of forgetting. Thus, if people learn to self-assess and prioritize information that will need to be remembered or have negative consequences if forgotten, the recall of said important information can be enhanced via strategic metacognitive mechanisms (cf. Koriat et al., 2006).

When engaging in these types of value/importance-based learning tasks, participants often demonstrate enhanced performance as the task endures (e.g., McGillivray & Castel, 2011). This improvement can be attributed to several key features of the paradigm, including feedback mechanisms and the intermixing of study–test blocks. Feedback plays a crucial role by informing participants about how well they performed on the task (either explicit feedback from experimenters or implicit feedback following participants’ monitoring of their output), which helps refine their memory strategies and adjust their focus on more important or valuable information. Including multiple study–test cycles allows participants to apply these adjustments almost immediately, thereby promoting iterative learning and strategy optimization. This process not only improves metacognitive awareness—by making learners more conscious of what they know and what they do not—but also enhances their ability to strategically allocate cognitive resources based on the perceived value of information. Gaining further experience through repeated encoding sessions is critical to developing and honing these adaptive strategies, suggesting that continued exposure and practice are essential for mastering the functions of responsible remembering.

Within the responsible memory framework, the effectiveness of value-directed remembering, which typically involves strategic planning and recruits top-down mechanisms (see Knowlton & Castel, 2022), contrasts with other memory-enhancing methods where rewards are tied to performance without the opportunity for strategic refinement. In tasks where cues denote potential rewards for future test performance, learners respond to the immediate motivational impact of potential rewards, which can enhance memory encoding and retention even in the absence of feedback or multiple study–test cycles. This suggests that while strategic, iterative adjustments in response to feedback are potent enhancers of memory, the motivational salience of rewards alone can also directly influence memory processes. The presence of reward cues can automatically trigger enhanced attention and deeper encoding of associated items, showcasing another facet of responsible remembering where the brain prioritizes information that has a higher perceived value, albeit through less consciously strategic means.

Promoting responsible remembering and prioritizing memory for important information can help avoid the negative consequences of forgetting. However, how a learner’s goals are framed can also impact how they remember. For example, Murphy and Knowlton (2022) presented learners with words paired with point values (i.e., a value-directed remembering procedure) but either framed participants’ goals and feedback in terms of gains or losses (see Murphy, Castel et al., 2024a, for a test of this procedure with older adults). Specifically, some participants were told to try to maximize their scores while others were told to try to minimize losses (and their feedback was either framed in terms of the number of points they gained via remembering or the number of points they lost due to forgetting). As a result of these differences in the framing of learners’ goals, the participants whose goals were framed in terms of losses were *less* selective towards high-value words than participants aiming to maximize gains. As such, participants aiming to minimize losses may have overextended their memory by trying to remember more rather than selectively focusing on the most important information. Thus, responsible rememberers should maximize the benefits of remembering important information (which should also result in avoiding consequences for forgetting) by selecting and remembering the most valuable information rather than avoiding forgetting by increasing the quantity of information remembered.

As demonstrated by participants’ superior memory for important information or information with consequences if forgotten in various situations, when presented with more information than can be remembered, participants engage in responsible remembering. In addition to the strategic allocation of study time when evaluating the importance of remembering, other cognitive mechanisms also contribute to responsible remembering. Specifically, responsible remembering may arise as a result of cognitive mechanisms such as reflecting on the importance of the information, useful forgetting, efficient attentional processes, and strategic retrieval operations employed by the learner (see Fig. 1 for a diagram of the responsible remembering process).

### Metacognitive reflectivity

As discussed in the previous section, *responsible remembering* encompasses metacognitive processes and the strategic allocation of attention toward important information to avoid consequences for forgetting. However, when metacognitive assessments fail to accurately predict memory performance, participants’ allocation of cognitive resources may be ineffectively used, leading to insufficient learning and failure to recall the information on a subsequent test (e.g., Hargis & Castel, 2019; Rhodes & Castel, 2009). Thus, in circumstances with negative consequences for poor metacognition and memory performance, responsible rememberers should reflect on the costs and benefits of later remembering information of varying value or importance and increase resolution by remembering the important information they expect to remember and being aware of what is less likely to be remembered (e.g., McGillivray & Castel, 2011).

Since the accuracy of JOLs can be influenced by many factors, researchers have investigated many different methods of judging future remembering and the accuracy of these predictions, such as binary JOLs (e.g., Hanczakowski et al., 2013; McGillivray & Castel, 2011), judgments of forgetting (e.g., Finn, 2008; Koriat et al., 2004), judgments of remembering and knowing (e.g., McCabe & Soderstrom, 2011), judgments of the ease of learning (e.g., Leonesio & Nelson, 1990), feeling of knowing judgments (e.g., Nelson, 1984), and judgments of retention (e.g., Tauber & Rhodes, 2012). Although each of these assessments captures different contributions to the accuracy of metacognition, these judgments do not incorporate item importance and can lead to a metacognitive disconnect between judgments and recall of important information (see Murphy & Castel, 2021a; Murphy, Hoover, et al., 2023c). Rather, reflecting on the importance of information may increase the recallability of this information (Ariel et al., 2009; Castel et al., 2012).

In contrast to more passive metacognitive assessments, judging the importance of remembering something, or making a *judgment of importance* (JOI), can be more useful and exemplify the notion of responsible remembering (Murphy & Castel, 2021a; Murphy, Hoover et al., 2023c). Specifically, JOIs may result in what I am calling *reflectivity*, a useful form of metacognitive reactivity (when making a judgment influences what is remembered; see Double & Birney, 2019; Double et al., 2018; Janes et al., 2018; Mitchum et al., 2016; Murphy, Halamish et al., 2023a; Murphy, Rhodes et al., 2024e; Soderstrom et al., 2015; Spellman & Bjork, 1992), whereby memory is enhanced for information judged as important to remember. For example, if a learner is asked to reflect on the importance of remembering something or the consequences of forgetting it, they may be more likely to allocate attention to this information and be more likely to remember it.

In the agenda-based regulation framework of study allocation decisions, learners partake in metacognitive monitoring to evaluate whether they have learned a piece of information and allocate study time accordingly (Dunlosky & Hertzog, 1997; Kornell & Metcalfe, 2006; Metcalfe & Kornell, 2005; Nelson et al., 1994). For example, Ariel et al. (2009) manipulated reward structure and item difficulty to examine how these factors influence learners’ decisions to select items for further study. Results demonstrated that learners’ agendas, shaped by the reward structure of the task, influenced study time allocation, often overriding the effects of item difficulty. This highlights that potential rewards can drive study behavior more strongly than the monitoring of intrinsic cues like item difficulty. Thus, rather than indicating the likelihood of remembering, asking participants how important it is to remember information may inform agendas and lead to better memory of important information.

Reflecting on the importance of information can subsequently lead to the selection of important information to remember. Specifically, as a product of having rated information as important to remember, the process of making JOIs could inform agendas and change the goal orientation process (e.g., Ariel et al., 2009) leading to subsequent reflectivity (rather than reactivity whereby memory is enhanced as a result of making a judgment) whereby participants demonstrate enhanced recall of important information as a result of making JOIs. Thus, there is an important metacognitive component to responsible remembering whereby remembering important information involves the identification and selection of valuable information.

Following the identification and selection of valuable information, learners engage in the selective rehearsal of the targeted to-be-remembered information (the most important information). For example, Hennessee et al. (2019) explored the impact of value on encoding strategies using a value-directed remembering procedure. Results revealed that participants allocated more cognitive resources and employed more elaborate and distinctive encoding strategies for high-value words. Thus, mechanisms like active rehearsal likely contribute to responsible remembering by ensuring that information deemed important is more deeply encoded, making subsequent retrieval more likely, particularly for information that carries greater consequences if forgotten.

Similar to high-value information, important information may prompt deeper semantic processing where individuals not only encode the basic features of the information but also relate it to other knowledge, increasing its integration into their cognitive schema. Additionally, the use of unique mnemonic aids, such as creating vivid mental images or forming acronyms, can increase the likelihood that important information is accessible when needed. These enhanced encoding strategies make the retrieval process more efficient, as the distinct and deeply encoded information can be accessed more readily and reliably. This enhanced retrievability is especially crucial in high-stakes situations where quick and accurate recall of important information is necessary, demonstrating how the importance of information can directly shape cognitive processes to optimize responsible remembering. However, there may be situations where it may be advantageous not to remember certain information. Specifically, deciding not to employ elaborate mnemonic strategies for information that is not important or relevant to one’s goals can also be considered a form of responsible remembering.

## Responsible forgetting

While recalling valuable information may be a sign of responsible remembering, forgetting is also a critical component of a functional memory system (see Storm, 2011). For example, remembering where you parked your car last week is not very helpful for finding your car today. Similarly, remembering old phone numbers may interfere with the recall of current ones. These instances of memory for old information exemplify the disadvantages of remembering information that is no longer useful—forgetting unimportant or outdated information can reduce interference for target information (see Murphy & Castel, 2022a, 2023b, for the effects of interference in value-directed remembering tasks). Thus, when presented with information of varying importance, to avoid forgetting important items, people may have developed the ability to forget information they do not need to remember so they can focus on the most important information and reduce competition for target information (cf. Anderson et al., 1994; Bjork et al., 1998; Fawcett & Hulbert, 2020). As a result, situations in which forgetting serves a functional purpose exemplify the need for goal-directed forgetting.

As evidence of the benefits of forgetting, directed forgetting tasks present participants with to-be-remembered as well as to-be-forgotten information. For example, in the seminal study by Bjork et al. (1968), participants were presented with two verbal items in a sequence and were instructed to forget the first item immediately before the presentation of the second. This process, known as directed forgetting, was investigated to see if it could reduce proactive interference from the first item on the memory of the second. Results indicated that recall of the second item was better when the forget instruction was given, demonstrating the effectiveness of directed forgetting in enhancing memory performance by reducing interference. Thus, forgetting certain information can benefit memory for other information, and this may be a form of responsible remembering.

Again, directed forgetting tasks demonstrate that memory for to-be-remembered information tends to be enhanced while memory for to-be-forgotten information tends to be impaired relative to controls (Basden & Basden, 1998; Bjork & Bjork, 1996; Bjork, 1998; MacLeod, 1998; Murphy & Castel, 2022c). Additionally, previous work indicates that when to-be-remembered and to-be-forgotten information is paired with point values, it may be more difficult to forget high-value items (Hennessee et al., 2019). Thus, forgetting certain information can benefit memory for target items, and when attempting to remember large amounts of information, responsible rememberers should prioritize recall for the most important items or information with the biggest consequences if forgotten while also forgetting unimportant items. Specifically, forgetting items that do not need to be remembered may facilitate memory for more items that do need to be remembered. This more efficient utilization of memory is a form of responsible remembering termed *responsible forgetting* (Murphy & Castel, 2021b).

**Fig. 4:**
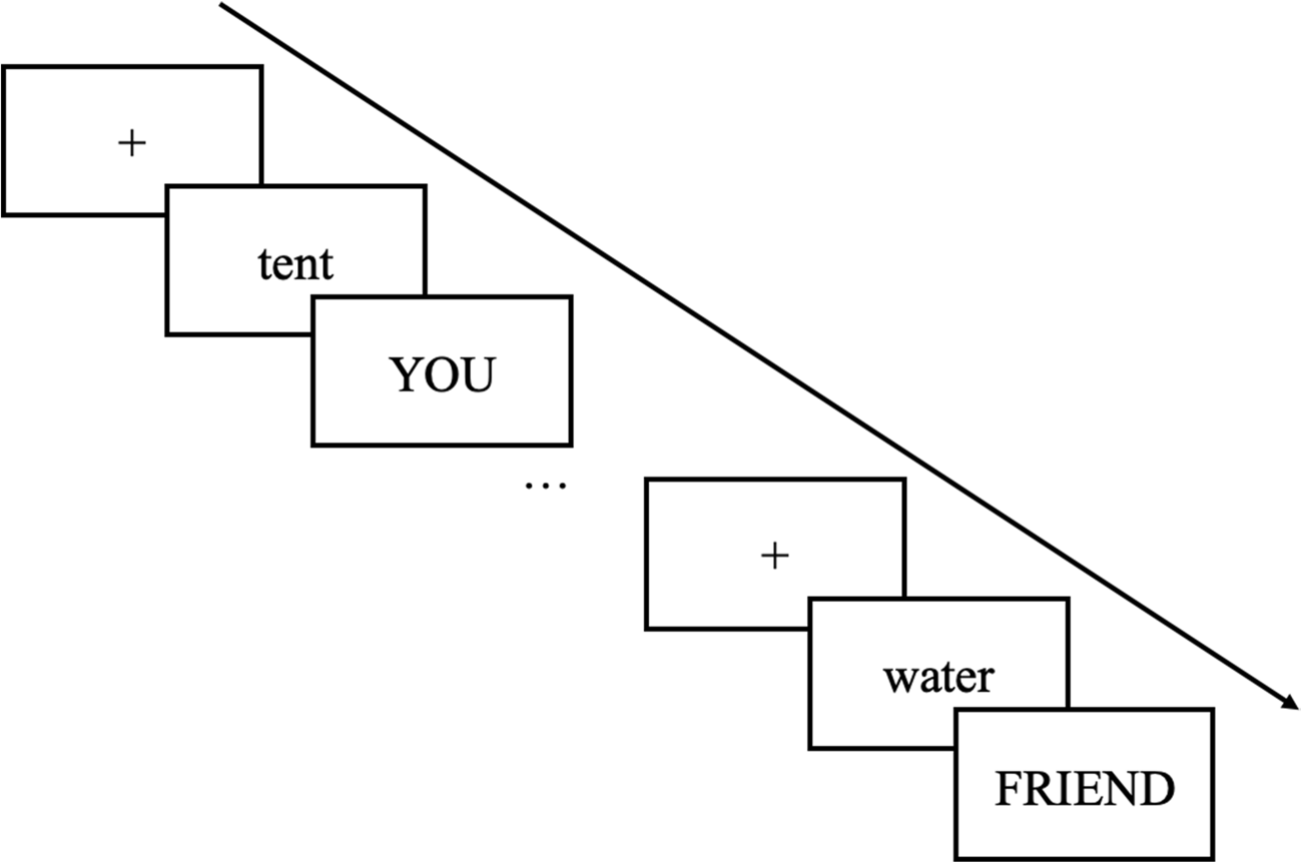
Example of the study phase in Murphy and Castel (2021b, 2022a)

Responsible forgetting posits that forgetting less important information or items that do not need to be remembered facilitates the retrieval of important, goal-relevant information by reducing competition for target information. To demonstrate responsible forgetting, Murphy and Castel (2021b) used an item-method directed forgetting paradigm whereby participants were presented with a list of to-be-remembered words with each word followed by a cue indicating whether the participant (“You”) or their hypothetical (“Friend”) was responsible for remembering the word (see Fig. 4). When asked to recall all the words, regardless of who was responsible for remembering them, participants best recalled and recognized items they were responsible for remembering. Thus, participants appeared to employ a strategy to maximize the total number of items accessible (they could not remember all the items themselves) by selectively remembering the items they were responsible for remembering and relying on their friend to remember the other items, perhaps attempting to forget these items. This suggests that learners may be able to harness the benefits of directed forgetting such that intentionally forgetting less goal-relevant information (items your friend will remember) could facilitate memory for target information.

Critically, Murphy and Castel (2021b) also demonstrated that participants’ recall and recognition were sensitive to importance, regardless of who was responsible for remembering each item. This behavior would be especially responsible in the case of an untrustworthy friend such that critical information may not be remembered if a forgetful friend was responsible for remembering it. Thus, to prevent consequences for forgetting, responsible rememberers should be tuned to remember important information even if this important information could be accessed via a different memory source. As such, both responsible remembering and responsible forgetting play important roles in the strategic control of the remembering process, and responsible rememberers should use their own memory to remember important information to reduce potential consequences for forgetting.

It is important to distinguish between forgetting and responsible for forgetting. When learners prioritize the encoding of valuable information, their focused attention on high-value items consequently diverts resources away from less valuable information. This often results in minimal encoding of lower-value information, which may subsequently become difficult or impossible to retrieve, leading to forgetting. In contrast, responsible forgetting involves the deliberate forgetting of information that is less relevant to one’s goals to benefit memory for important information. For instance, in the responsible forgetting paradigm (Murphy & Castel, 2021b), learners initially view and process each item before a cue indicates whether they or a hypothetical friend are responsible for remembering it. If the cue designates the learner as responsible, they are likely to engage in further rehearsal of the item, enhancing its retention (see Basden & Basden, 1998, for accounts of item-method directed forgetting). However, if the cue indicates that the responsibility lies with the friend, a responsible rememberer would strategically halt further rehearsal of that item or even engage in the suppression of that information. The subsequent forgetting of these items conserves cognitive resources for target information, leading to more responsible remembering.

Similar to the benefits of directed forgetting, previous work has demonstrated that offloading some to-be-remembered information (e.g., using tools like a computer or a notepad to assist memory) facilitates memory for other to-be-remembered information by reducing the interference from offloaded information when recalling target information (Dror & Harnad, 2008; Henkel, 2014; Risko & Dunn, 2015; Risko & Gilbert, 2016; Sparrow et al., 2011; Storm & Stone, 2014). Thus, offloading may be a tool for responsible rememberers to further maximize the accessibility of important information. Specifically, offloading could function similarly to responsible forgetting if people strategically choose not to use memory capacity for less-important information, thereby freeing up cognitive resources to enhance the retention of the most crucial information. Alternatively, given the fallibility of memory, offloading the most valuable information may be a more reliable way to ensure access to important information.

Whether relying on a friend to remember something or using an external store like a cell phone, offloading has many obvious benefits (e.g., if the external store is more reliable than memory, the likelihood that the information will be accessible when needed is increased), but there are also drawbacks. For example, if offloaded information becomes unavailable (e.g., dead phone battery), memory for that information is impaired (e.g., Eskritt & Ma, 2014; Kelly & Risko, 2019a, 2019b; Lu et al., 2020; Marsh & Rajaram, 2019). Additionally, if the offloaded information was important, the forgetting of this information could lead to negative consequences (e.g., your friend forgetting water for the camping trip or forgetting about a meeting with your boss that you had written on your phone). Thus, overreliance on offloading may be detrimental to responsible remembering.

Previous work has found that learners forgo selective memory processes when allowed to rely on an external store. For example, Park et al. (2022) examined how reliance on an external memory store affects the recall of high- and low-value information. Participants were presented with words associated with different point values and were informed in some trials that they could rely on an external store as a memory aid while in other trials they were informed that they could not. Results revealed that the recall advantage for high-value information was significantly diminished when participants expected access to an external store. This suggests that reliance on external memory aids can reduce the selective encoding and recall of valuable information. Thus, learners appear to prefer offloading rather than using selective memory for responsible remembering, and the dangers of offloading can lead to negative consequences for forgetting.

Although people may disengage responsible remembering when everything can be offloaded, it is often impractical to offload all information that will be needed later. Rather, people use external stores to save subsets of information, and this may have benefits for responsible remembering. For example, Murphy (2023b) investigated how learners choose to offload information of various values when the external store has limited capacity (we are usually not able to offload everything we need to remember so we need to be selective) and how the unexpected unavailability of offloaded information affects memory performance. Participants were presented with words paired with point values (a typical value-directed remembering procedure), and participants were allowed to offload some of the words. Results indicated that learners selectively offloaded high-value words, which could be an optimal strategy if the external store is more dependable than memory. However, when the offloaded words were unexpectedly unavailable, learners frequently failed to recall the high-value words, highlighting the potential risks of offloading. Thus, while selectively offloading valuable information can enhance responsible remembering if one has access to the external store, responsible rememberers should selectively encode valuable information—even if it could be offloaded—to prevent the consequences of forgetting if the external store becomes unavailable.

Together, the present evidence suggests that both forgetting and offloading can contribute to responsible remembering. Specifically, while responsible forgetting involves the selective elimination of less critical information, strategic offloading serves as an extension of responsible forgetting where valuable information is intentionally stored externally rather than being forgotten. This allows people to free up cognitive resources while still maintaining access to important information, thereby tapping into the benefits of responsible forgetting by ensuring that memory capacity is optimally used without losing access to crucial information. Thus, both strategically offloading important information and selectively forgetting less important information may be a critical aspect of a functional memory system.

### Responsible attention

In addition to responsible forgetting, there is likely an attentional component to the strategic remembering of important information. For example, there are detrimental effects of divided attention on encoding and later memory (Castel & Craik, 2003; Craik et al., 1996; Naveh-Benjamin et al., 2000), and reduced attentional recourses during encoding generally impairs selectivity for important information (see Elliott & Brewer, 2019; Murphy & Castel, 2023c; Murphy, Schwartz et al., 2024d; Siegel et al., 2021; but see Middlebrooks et al., 2017; Siegel & Castel, 2018). Thus, the availability of attentional resources during encoding can affect learners’ ability to execute goal-oriented agendas, and a full allotment of attentional resources may be necessary to engage in responsible remembering via strategic encoding mechanisms.

With a full allotment of attentional resources, learners should be able to metacognitively reflect on what information is most important, select it for study, and later retrieve this information to avoid the consequences of forgetting. However, conditions where the learners’ attention is divided could disrupt some of these processes, potentially leading to the forgetting of valuable information. Thus, there may be a nuanced impact of attentional resources on responsible remembering such that a full allotment of attentional resources is necessary for some aspects of responsible remembering, but reduced attention may still be sufficient for achieving success in other aspects. This potential complex interplay where the level of attention required can vary depending on the specific demands of the memory task illustrates *responsible attention*: the deliberate allocation and prioritization of cognitive resources towards important information during the responsible remembering process. This strategic focus ensures that valuable information is effectively processed and encoded, enhancing overall memory accuracy and utility in situations where attentional resources may be limited or divided.

To investigate the role of attentional resources in responsible remembering, Murphy and Castel (2023c) examined participants’ memory for valuable information, metacognitive accuracy, and cognitive control mechanisms when under full and divided attention. If participants are unable to selectively remember valuable information, accurately monitor their learning, or execute goal-directed cognitive control when under divided attention, this would indicate that attentional recourses are a key component of responsible remembering. However, although some functions may be impaired when attention is divided, some of the learners’ cognitive abilities contributing to responsible remembering may be preserved.

In their first experiment, Murphy and Castel (2023c) utilized a value-directed remembering paradigm with a metacognitive “betting” component (similar to McGillivray & Castel, 2011) with participants either under full or divided attention. Specifically, participants could only score points if they “bet” on a word and recalled it—if they bet on a word and failed to recall it, those points were subtracted from their score. Results revealed that participants’ betting decisions were less sensitive to value when their attention was divided, suggesting that full attention may be necessary for the selection process of responsible remembering. Additionally, memory selectivity was impaired under divided attention (consistent with prior work, see Elliott & Brewer, 2019; Murphy, Schwartz et al., 2024d; Siegel et al., 2021; but see Middlebrooks et al., 2017; Siegel & Castel, 2018). Thus, a full complement of attention is necessary for selecting as well as encoding important information to remember, indicating that learners’ awareness of the consequences of forgetting may be impaired with fewer attentional recourses available during encoding.

Next, Murphy and Castel (2023c) presented participants with unassociated word pairs and solicited metacognitive predictions of later recall (JOLs) for each pair. Results revealed that the relative accuracy of participants’ metacognitive judgments was preserved or may even be enhanced when studying under divided attention (see also Peng & Tullis, 2021). Given this preservation of metacognitive accuracy in Experiment 2 but the impaired metacognitive “betting” decisions in Experiment 1, this suggests that full attention is not necessary for the ability to identify what will be remembered and what will be forgotten but learners’ ability to reflect on the importance of remembering an item or the consequences of forgetting it and making corresponding metacognitive control decisions is impaired under divided attention.

Finally, Murphy and Castel (2023c) used the responsible forgetting paradigm (see Murphy & Castel, 2021b) where participants are presented with a list of words to pack for a camping trip with either the participant or a hypothetical friend responsible for remembering each item. Results revealed that participants’ ability to strategically prioritize goal-relevant information (the items they were responsible for remembering) at the expense of information that could be offloaded (the items their friend was responsible for remembering) was preserved under divided attention. However, an additional analysis provided some evidence that participants’ ability to prioritize important items that they were responsible for remembering was impaired under divided attention. This further suggests that while participants may still be able to engage in strategic forgetting of important information with fewer attentional resources, full attention is needed to prioritize memory for important items.

While full attention may be necessary for the selection and encoding of important information, partial attention may be sufficient for the retrieval of this information. To test the importance of having full attention at retrieval, Murphy, Schwartz et al. (2024d) presented participants with lists of to-be-remembered items of various subjective and objective value, and either divided their attention during encoding or retrieval (participants were asked to complete a secondary task while simultaneously encoding or recalling the words). Divided attention disrupted selective memory (i.e., better recall for high- relative to low-value items) during encoding but not retrieval. Thus, although limited attentional resources during encoding may impair responsible remembering, the ability to recall important information can be maintained with fewer attentional resources.

This trend of selective memory being less susceptible to the effects of divided attention at retrieval compared with encoding may be due to the nature of cognitive processes involved at each stage. During encoding, attention is crucial for the initial processing and selection of information according to learners’ agendas (Ariel et al., 2009). Thus, with fewer attentional resources, learners are less able to identify which items to focus on and subsequently engage in selective rehearsal (as well as other more elaborative encoding strategies) for these items. In contrast, retrieval processes often rely on cues and previously established memory connections that can be activated even under conditions of reduced attentional capacity. This suggests that once information has been successfully encoded, the cues used to retrieve it do not require the same level of focused attention as the initial encoding. Therefore, strategic retrieval can be more robust against dual-task interference, making it possible to access important information even in attentionally demanding situations.

Together, this evidence suggests that a full allotment of attentional resources available during encoding is necessary for learners to reflect on the importance or value of incoming information and subsequently execute goal-based strategies to selectively encode this information, though reduced attentional resources are still sufficient for some cognitive abilities such as accurately monitoring learning and certain aspects of responsible forgetting. This illustrates the concept of *responsible attention* such that there is an attentional component to responsible remembering whereby full attention is needed to engage in strategic metacognitive and selective encoding processes. Thus, within the framework of responsible remembering, responsible attention serves not as an independent process, but as a crucial facilitative mechanism that underpins both the remembering and forgetting of information. This conceptualization underscores the importance of allocating full attentional resources to the strategic encoding of important information and the forgetting of less valuable information. To achieve responsible remembering, having a full allotment of attentional recourses is paramount for the identification and selection of important information to remember, though full attention may not be necessary during retrieval.

### Responsible retrieval

In addition to responsible forgetting and responsible attention, responsible remembering also involves *responsible retrieval*: the use of strategic retrieval operations to selectively remember important information. For example, in value-directed remembering tasks where participants are presented with words paired with point values counting towards their scores if recalled, participants are most likely to initiate recall with valuable words (Murphy & Castel, 2021c; Murphy, Schwartz et al., 2024d; Stefanidi et al., 2018). Additionally, participants strategically organize retrieval by recalling goal-relevant or valuable items before less relevant or low-value information (see Murphy & Castel, 2021b, 2021c). Thus, one strategy learners may employ to remember responsibly is to proceed in a most important to least important order of recall to ensure efficient retrieval/performance.

Recalling valuable or goal-relevant information before less important information may result in ideal memory outcomes as a consequence of reduced output interference: the decreased recall probability as a function of later output position (Bäuml, 1998; Roediger, 1974; Roediger & Schmidt, 1980; Smith, 1971, 1974). Specifically, if low-value information is recalled first, important information may suffer from the costs of output interference. Conversely, if target information is recalled first, the information suffering from output interference will primarily be low-value information. Thus, responsible remembering encompasses retrieval strategies to reduce output interference and facilitate the recall of goal-relevant items.

Strategic retrieval strategies may be crucial for engaging in responsible remembering when conditions favor recall patterns not well suited for selective memory. For example, people often rely on habitual, serial processing such as reading from left to right when studying information. Murphy, Schwartz et al. (2022b) tested whether learners could overcome this serial processing by presenting participants with lists of word triads (e.g., “troop alley pedal”). Participants were told that following the presentation of each triad, a cue would indicate which word was the most valuable. When the learners’ goal was simply to maximize total recall, they engaged in serial remembering (i.e., recall was guided by an item’s location within the study phase) but when a cue indicated that a word was more valuable than its neighbors, participants selectively remembered these words and overcame the tendency to engage in serial remembering (i.e., recall was guided by value). Thus, to maximize memory utility, learners should override habitual processes and engage in strategic memory processes.

In addition to strategic retrieval operations, responsible retrieval also involves selective memory for important information in conditions detrimental to overall memory performance. For example, Murphy, Schwartz et al. (2024d) demonstrated that divided attention during retrieval did not impair memory selectivity. Rather, during the recall phase, participants still initiated recall with the most important information and were selective for valuable information despite being required to complete a secondary task. Thus, while a reduced allotment of attentional resources during encoding can reduce responsible remembering (see responsible attention section above), the ability to engage in strategic retrieval operations and recall information of importance can be preserved in more difficult retrieval conditions.

Furthermore, Hoover et al. (2024) presented participants with words paired with point values and either gave participants sufficient time or only a few seconds to recall the words from each list. Results revealed that rushed participants were more selective than participants with ample time, indicating that some conditions that are detrimental to overall recall can enhance one’s ability to focus on retrieving valuable information (see also Middlebrooks, Murayama, et al., 2016b). Thus, responsible remembering involves not simply the strategic allocation of attention during encoding, but also strategic retrieval operations like recalling the most important or valuable items before less important ones and selectively remembering valuable information in the face of competing factors.

### Older adults as responsible rememberers

As depicted in Fig. 1, responsible remembering is influenced by several interrelated cognitive functions including metacognitive reflectivity, responsible forgetting, responsible attention, and responsible retrieval. As a result, individual variation in these abilities is likely to predict differences in how effectively individuals engage in responsible remembering. For example, Robison and Unsworth (2017) demonstrated that individuals with greater working memory capacity are better able to engage in strategic value-based memory (see also Elliott, McClure et al., 2020b; Griffin et al., 2019; Murphy et al., 2021). Thus, individual differences may influence responsible remembering (Underwood, 1975) and since older adults often experience various cognitive deficits (cf. Craik & Bosman, 1992; Hess, 2005; Park & Festini, 2017; Salthouse, 2010), there may be important differences in how younger and older adults remember important information.

Engaging in responsible remembering is especially important for older adults due to their more limited cognitive resources and the need to use them strategically. As a result of having experienced more instances of memory failure, older adults may have learned to engage in an adaptive realization of what can be successfully remembered and recruit these metacognitive insights to selectively remember important information (see Whatley et al., 2021). For example, task experience and interventions can update learning in older adults (e.g., Friedman et al., 2015; McGillivray & Castel, 2011; Middlebrooks, McGillivray, et al., 2016a) such that instances of forgetting can help older adults learn to prioritize high-value information and selectively remember certain associations if they are likely to be important later (e.g., Friedman et al., 2015; McGillivray & Castel, 2011; Middlebrooks, McGillivray, et al., 2016a). This can take the form of learning to remember important medication warnings (e.g., Hargis & Castel, 2018a, 2018b), remembering the names and faces of people that one cares about (e.g., Hargis & Castel, 2017), or learning vocabulary terms of a new language (Murphy, Hargis et al., 2023b).

As initial evidence of older adults as responsible rememberers, early work demonstrated that older adults can remember information paired with high point values as well as younger adults by selectively prioritizing and encoding these items (e.g., Castel et al., 2002, 2007, 2013; see Murphy et al., in press; Murphy, Hoover et al., 2024c, for an examination of age-related similarities and differences in the automatic and strategic effects of value). Additionally, older adults can learn to become more responsible rememberers when there are consequences for forgetting by implementing strategies that lead to more accurate metacognition and better memory for high-value items (e.g., McGillivray & Castel, 2011). Thus, in some instances, older adults may have adapted to be more responsible rememberers than younger adults such that older adults more efficiently use their cognitive resources by being more strategic in their memory processes.

To compare memory performance in a situation demanding responsible remembering, Murphy, Hoover et al. (2024c) presented younger and older adults with children they would hypothetically be babysitting, each with three preferences: a food they like, a food they dislike, and a food they are allergic to and must avoid (similar to Murphy & Castel, 2021a; Murphy, Hoover et al., 2023c; see Fig. 3). Consistent with older adults being more responsible rememberers, results showed that older adults were better than younger adults at prioritizing children’s allergies relative to the other preferences, and participants’ tendency to selectively remember the allergies increased with task experience. These results suggest that older adults can employ strategies that enhance the recall of important information, exemplifying responsible remembering.

In addition to using memory, offloading information to external stores may be a tool that can enhance responsible remembering. For example, Murphy and Castel (2023a) investigated how the objective and subjective value or importance of information influences the offloading decisions of younger and older adults. Participants were presented with lists of items (either lists of unassociated words paired with objective point values that would contribute to participants’ scores if recalled or lists of associated items that differed in subjective value, such as a list of items to pack for vacation) and were allowed to offload a subset of the presented items. When words were paired with objective point values, younger adults were more selective in their offloading decisions and subsequent recall than older adults, showing a greater tendency to offload and recall high-value items than low-value items relative to older adults. In contrast, when the items differed in subjective value, older adults were more selective in their offloading decisions than younger adults, preferring to offload words they considered important relative to items they considered less important. These differences in offloading tendencies suggest that older adults may better engage in responsible remembering by strategically using tools at their disposal to ensure the accessibility of important information to avoid consequences for forgetting.

In addition to age-related differences in selecting and remembering important information, younger and older adults may also differ in the capacity to strategically forget less important information to benefit memory for target information. To investigate age-related differences in responsible forgetting, Murphy and Castel (2022b) presented younger and older adults with a list of items to bring on a camping trip followed by a cue indicating whether participants or a hypothetical friend was responsible for remembering each item (similar to Murphy & Castel, 2021b; see Fig. 4). Both younger and older adults similarly engaged in responsible remembering and forgetting by better recalling and recognizing items they were responsible for remembering relative to their “friend,” indicating a tactical utilization of their reduced memory capacity.

Responsible forgetting enhances cognitive efficiency by reducing cognitive load, which may be particularly beneficial for older adults who already experience memory difficulties (e.g., Craik & Bosman, 1992). Specifically, in tasks involving responsible forgetting, the presence of rewards for remembering crucial information and consequences of forgetting critical items may serve to focus attention and enhance the use of cognitive resources. Thus, if learners prioritize memory for the important items they were assigned to remember and forget the items their friend will remember, this strategic allocation of resources towards the items the participants were responsible for remembering leads to a more selective information search during retrieval (less items are competing with important information at retrieval), ultimately contributing to responsible remembering in younger and older adults.

Although older adults’ ability to engage in responsible remembering can rival or even exceed that of younger adults in some cases, older adults’ ability to selectively remember valuable information can be reduced under some conditions. For example, when rushed during recall, older adults are similarly selective compared with when they are given sufficient recall time, but these conditions enhance selectivity in younger adults (Hoover et al., 2024). When facing time constraints during recall, the effectiveness of retrieval strategies becomes crucial for responsible remembering. In such scenarios, participants need to organize their output to prioritize the most valuable items as these items may not be retrieved if time runs out, even if they are accessible in memory. Thus, while recalling low-value items can still be beneficial, focusing on these items when time is limited is less advantageous, and evidence suggests that younger adults may be better than older adults at organizing their output to retrieve the most critical information first. In contrast, older adults may be less strategic in their output, unable to adapt to the demands of the task—they still recall valuable items before low-value items but do not strategically reorganize retrieval strategies to meet the demands of the task. Thus, the typically slower cognitive processing in older adults, combined with their reduced ability to quickly allocate attentional resources under time constraints, indicates that while older adults are capable of effectively employing responsible remembering and attention under normal conditions, their performance may be compromised in more demanding situations.

As further evidence that younger adults display superior responsible retrieval compared with older adults, Murphy and Castel (2024) presented younger and older adults with triads of words, with one word having a higher point value than the others (similar to the procedure from Murphy, Schwartz et al., 2022b, described above). Results revealed that younger adults demonstrated better strategic memory than older adults by better recalling high-value words and were more likely to initiate recall with a high-value word compared with older adults. Moreover, older adults were more likely to recall words in their presented order while younger adults strategically recalled successive high-value words. Thus, in some conditions, younger adults better prioritize high-value words using strategic encoding and retrieval processes whereas older adults rely more on habitual processes, indicating that responsible remembering can be volatile in both younger and older adults, with certain study and test conditions leading to differential remembering of important information.

Together, evidence suggests that both younger and older adults prioritize memory for important information with consequences for forgetting but there may be age-related differences in the mechanisms of responsible remembering. Specifically, older adults may be better than younger adults at identifying and selecting information that is important to remember. Thus, the experience of previous memory failures paired with the awareness of how much or how often one forgets may lead older adults to more efficiently allocate cognitive resources to engage in responsible remembering. As such, older adults focus primarily on remembering what is most critical to compensate for declines in memory (Friedman et al., 2015; Hargis & Castel, 2018b; McGillivray & Castel, 2017; Middlebrooks, McGillivray et al., 2016a).

Although both younger and older adults engage in responsible forgetting to reduce competition for target information, responsible retrieval is impaired in older adults. Specifically, younger adults are better able to organize retrieval to prioritize the output of important information. However, responsible remembering in older adults may still reflect a strategic and selective information search in the context of decision-making—using fewer pieces of information or searching less exhaustively but also focusing on more diagnostic information (e.g., Mata et al., 2007; Queen et al., 2012). Thus, relative to younger adults, older adults may recruit different cognitive mechanisms to engage in responsible remembering.

One theoretical framework fitting with older adults’ ability to recall important information is selective optimization with compensation (Baltes, 1997; Baltes & Baltes, 1990; Freund & Baltes, 2000). According to this framework, older adults experience various memory deficits and compensate for memory declines by adjusting their goals and expectations. In the context of responsible remembering, older adults strategically focus on important or goal-related information at the expense of less important or relevant information (e.g., Castel, 2008a; Siegel & Castel, 2018; see also Bäckman & Dixon, 1992; Baltes & Baltes, 1990). Furthermore, while younger adults generally report growth-oriented goals, older adults report greater maintenance or loss prevention goals (Ebner et al., 2006; Freund, 2008). Thus, the availability of cognitive resources may influence whether one’s goals are more focused on maximizing gains or minimizing losses (see Murphy, Castel et al., 2024a). In sum, while older adults’ memory impairments can prevent initial learning of important information and impair later remembering, older adults generally engage in the efficient and effective use of selective memory to remember important information and exemplify the need for *responsible remembering*.

## Conclusions, pending issues, and future directions

Focusing on remembering information that has the greatest consequences if forgotten is a potentially adaptive process called *responsible remembering*. Although responsible remembering typically deals with remembering*, metacognition, forgetting, attention,* and *retrieval* are all critical aspects as well. Specifically, the responsible remembering framework (see Fig. 1) involves not only selective memory processes but also the identification and selection of what to remember (metacognitive reflectivity), the forgetting of less important information to facilitate memory for items that do need to be remembered (responsible forgetting), the functional prioritization of attention at the expense of competing factors (responsible attention), and the selective recall of important information via efficient retrieval strategies (responsible retrieval).

Responsible remembering, forgetting, attention, and retrieval are highly interrelated functions that work in concert to optimize memory performance. Through the process of responsible remembering, individuals strategically identify, prioritize, and encode information that is deemed important. Responsible forgetting complements this by selectively forgetting less relevant information, thereby reducing cognitive load and enhancing focus on important information. Meanwhile, responsible attention underpins responsible remembering by allocating cognitive resources toward these memory processes. Additionally, responsible retrieval involves the initiation of retrieval with valuable information and organizing output according to information importance. Together, these functions form a cohesive system that aims to selectively prioritize, encode, and recall information that is deemed important based on its anticipated utility or the consequences of forgetting, and considering the importance of information may be a critical memory adaptation as we age.

The responsible remembering framework, which fits with adaptive memory views (see Nairne, 2010, 2013, 2015; Nairne & Pandeirada, 2008, 2010; Nairne et al., 2007), primarily focuses on the strategic aspects of memory, particularly how people manage their memory resources to prioritize information deemed most useful or important. However, this framework also aligns with other theories of adaptive memory that emphasize the role of reward in memory but differs in its emphasis on strategy use. For instance, prior work has shown that emotionally charged information is better remembered than neutral information (e.g., Buchanan et al., 2006; Doerksen & Shimamura, 2001; Kensinger & Corkin, 2003; Kleinsmith & Kaplan, 1963; LaBar & Phelps, 1998; Rubin & Friendly, 1986; see also Buchanan & Adolphs, 2002; Murphy & Isaacowitz, 2008). Unlike these findings, which often focus on automatic, affect-driven processes, the responsible remembering framework involves deliberate strategies (e.g., metacognitive reflectivity, responsible forgetting, and responsible retrieval). Furthermore, the responsible remembering framework also considers how repeated interactions with feedback and value can shape and refine memory strategies over time, an aspect less emphasized in work where a single exposure to a reward cue drives memory.

Although prior work has demonstrated responsible remembering in a variety of situations, future work should investigate responsible remembering in more consequential situations rather than relatively trivial laboratory tasks. For example, simulated negative consequences and the fear of forgetting may be taken less into account in experimental situations compared with real-world scenarios. Specifically, tasks offering participants more salient consequences for forgetting (such as losing money or social embarrassment) could further elucidate the strategic cognitive mechanisms of responsible remembering. Moreover, future research should work to further elucidate the underlying cognitive and neural mechanisms guiding effective responsible remembering behavior (see Cohen et al., 2014; Elliott, Blais, et al., 2020a; Elliott et al., 2022; Shigemune et al., 2014).

In addition to work examining memory processes when there are more salient negative consequences for forgetting, future work should investigate responsible remembering processes when learners are presented with potential rewards for remembering. Specifically, in real life, important information is often associated with a reward if remembered. For example, if a donut shop provides a free donut for students with favorable grades, remembering their report card would result in the reward of a free donut. However, there is not a direct consequence for forgetting the report card, only the lack of a small reward. Although value-directed remembering research has demonstrated memory for words paired with high point values (i.e., high “rewards”), future work should implement rewards for remembering like earning money or promoting social status rather than consequences for forgetting. Additionally, some people are highly motivated by rewards while others are more risk-averse, so individual differences and how responsible remembering relates to individual goals should be examined (see Murphy, Castel et al., 2024a; Murphy & Knowlton, 2022).

In sum, if younger and older adults learn to self-assess and prioritize what information will need to be remembered or have negative consequences if forgotten, engage in strategic forgetting, efficiently allocate their attentional resources, and utilize effective retrieval operations, the recall of said important information can be enhanced, a critical interaction between cognitive and metacognitive processes captured by *responsible remembering*.

## Footnotes

1 This review exclusively discusses item-level judgments of learning rather than global predictions of memory performance (e.g., the total number of items that will be remembered; e.g., Connor et al., 1997).

## References

[CR1] Anderson, J. R., & Schooler, L. J. (1991). Reflections of the environment in memory. *Psychological Science,**2*, 396–408.

[CR2] Anderson, J. R., & Schooler, L. J. (2000). The adaptive nature of memory. In E. Tulving & F. I. M. Craik (Eds.), *The Oxford handbook of memory* (pp. 557–570). Oxford University Press.

[CR3] Anderson, M. C., Bjork, R. A., & Bjork, E. L. (1994). Remembering can cause forgetting: Retrieval dynamics in long-term memory. *Journal of Experimental Psychology: Learning, Memory, and Cognition,**20*, 1063–1087. 7931095 10.1037//0278-7393.20.5.1063

[CR4] Anderson, R. B., Tweney, R. D., Rivardo, M., & Duncan, S. (1997). Need probability affects retention: A direct demonstration. *Memory & Cognition,**25*, 867–872. 9421573 10.3758/bf03211331

[CR5] Anselmi, N., Montaldo, S., Pomilla, A., & Maraone, A. (2020). Forgotten baby syndrome: Dimensions of the phenomenon and new research perspectives. *Rivista di psichiatra,**55*, 112–118. 10.1708/3333.33026 32202549

[CR6] Ariel, R. (2013). Learning what to learn: The effects of task experience on strategy shifts in the allocation of study time. *Journal of Experimental Psychology: Learning, Memory, and Cognition,**39*, 1697–1711. 23751010 10.1037/a0033091

[CR7] Ariel, R., & Dunlosky, J. (2013). When do learners shift from habitual to agenda-based processes when selecting items for study? *Memory & Cognition,**41*, 416–428. 23135748 10.3758/s13421-012-0267-4

[CR8] Ariel, R., Dunlosky, J., & Bailey, H. (2009). Agenda-based regulation of study-time allocation: When agendas override item-based monitoring. *Journal of Experimental Psychology: General,**138*, 432–447. 19653800 10.1037/a0015928

[CR9] Bäckman, L., & Dixon, R. A. (1992). Psychological compensation: A theoretical framework. *Psychological Bulletin,**112*, 259–283. 1454895 10.1037/0033-2909.112.2.259

[CR10] Ball, H. B., Klein, K. N., & Brewer, G. A. (2014). Processing fluency mediates the influence of perceptual information on monitoring learning of educationally relevant materials. *Journal of Experimental Psychology: Applied,**20*, 336–348. 25347408 10.1037/xap0000023

[CR11] Baltes, P. B. (1997). On the incomplete architecture of human ontogeny: Selection, optimization, and compensation as foundation of developmental theory. *American Psychologist,**52*, 366–380. 9109347 10.1037//0003-066x.52.4.366

[CR12] Baltes, P. B., & Baltes, M. M. (1990). Psychological perspectives on successful aging: The model of selective optimization with compensation. In P. B. Baltes & M. M. Baltes (Eds.), *Successful aging: Perspectives from the behavioral sciences* (pp. 1–34). Cambridge University Press.

[CR13] Basden, B. H., & Basden, D. R. (1998). Directed forgetting: A contrast of methods and interpretations. In J. M. Golding & C. M. MacLeod (Eds.), *Intentional forgetting: Interdisciplinary approaches* (pp. 139–172). Erlbaum.

[CR14] Bäuml, K. (1998). Strong items get suppressed, weak items do not: The role of item strength in output interference. *Psychonomic Bulletin & Review,**5*, 459–463.

[CR15] Besken, M., & Mulligan, N. W. (2013). Easily perceived, easily remembered? Perceptual interference produces a double dissociation between metamemory and memory performance. *Memory & Cognition,**41*, 897–903. 23460317 10.3758/s13421-013-0307-8

[CR16] Bjork, R. A. (1998). Intentional forgetting in perspective: Comments, conjectures, and some directed remembering. In J. M. Golding & C. M. MacLeod (Eds.), *Intentional forgetting: Interdisciplinary approaches* (pp. 453–481). Erlbaum.

[CR17] Bjork, E. L., & Bjork, R. A. (1996). Continuing influences of to-be-forgotten information. *Consciousness and Cognition,**5*, 176–196. 8978530

[CR18] Bjork, R. A., LaBerge, D., & Legrand, R. (1968). The modification of short-term memory through instructions to forget. *Psychonomic Science,**10*, 55–56.

[CR19] Bjork, E. L., Bjork, R. A., & Anderson, M. C. (1998). Varieties of goal directed forgetting. In J. M. Golding & C. M. MacLeod (Eds.), *Intentional forgetting: Interdisciplinary approaches* (pp. 103–137). Erlbaum.

[CR20] Broder, A., & Undorf, M. (2019). Metamemory viewed through the judgment lens. *Acta Psychologica,**197*, 153–165. 31158737 10.1016/j.actpsy.2019.04.011

[CR21] Buchanan, T. W., & Adolphs, R. (2002). The role of the human amygdala in emotional modulation of long-term declarative memory. In S. C. Moore & M. Oaksford (Eds.), *Emotional cognition: From brain to behaviour* (pp. 9–34). Benjamins.

[CR22] Buchanan, T. W., Etzel, J. A., Adolphs, R., & Tranel, D. (2006). The influence of autonomic arousal and semantic relatedness on memory for emotional words. *International Journal of Psychophysiology,**61*, 26–33. 16427713 10.1016/j.ijpsycho.2005.10.022

[CR23] Busey, T. A., Tunnicliff, J., Loftus, G. R., & Loftus, E. F. (2000). Accounts of the confidence-accuracy relation in recognition memory. *Psychonomic Bulletin & Review,**7*, 26–48. 10780019 10.3758/bf03210724

[CR24] Castel, A. D. (2008a). The adaptive and strategic use of memory by older adults: Evaluative processing and value-directed remembering. In A. S. Benjamin & B. H. Ross (Eds.), *The psychology of learning and motivation* (vol. 48, pp. 225–270). Academic Press.

[CR25] Castel, A. D. (2008b). Metacognition and learning about primacy and recency effects in free recall: The utilization of intrinsic and extrinsic cues when making judgments of learning. *Memory & Cognition,**36*, 429–437. 18426071 10.3758/mc.36.2.429

[CR26] Castel, A. D., & Craik, F. I. M. (2003). The effects of aging and divided attention on memory for item and associative information. *Psychology and Aging,**18*, 873–885. 14692872 10.1037/0882-7974.18.4.873

[CR27] Castel, A. D., Benjamin, A. S., Craik, F. I. M., & Watkins, M. J. (2002). The effects of aging on selectivity and control in short-term recall. *Memory & Cognition,**30*, 1078–1085. 12507372 10.3758/bf03194325

[CR28] Castel, A. D., Farb, N. A. S., & Craik, F. I. M. (2007). Memory for general and specific value information in younger and older adults: Measuring the limits of strategic control. *Memory & Cognition,**35*, 689–700. 17848027 10.3758/bf03193307

[CR29] Castel, A. D., McGillivray, S., & Friedman, M. C. (2012). Metamemory and memory efficiency in older adults: Learning about the benefits of priority processing and value-directed remembering. In M. Naveh-Benjamin & N. Ohta (Eds.), *Memory and aging: Current issues and future directions* (pp. 245–270). Psychology Press.

[CR30] Castel, A. D., Murayama, K., Friedman, M. C., McGillivray, S., & Link, I. (2013). Selecting valuable information to remember: Age-related differences and similarities in self-regulated learning. *Psychology and Aging,**28*, 232–242. 23276210 10.1037/a0030678

[CR31] Chang, M., & Brainerd, C. J. (2022). Association and dissociation between judgments of learning and memory: A Meta-analysis of the font size effect. *Metacognition and Learning,**17*, 443–476. 35250403 10.1007/s11409-021-09287-3 PMC8883023

[CR32] Cohen, M. S., Rissman, J., Suthana, N. A., Castel, A. D., & Knowlton, B. J. (2014). Value-based modulation of memory encoding involves strategic engagement of fronto-temporal semantic processing regions. *Cognitive, Affective, & Behavioral Neuroscience,**14*, 578–592. 10.3758/s13415-014-0275-x PMC4074434 24683066

[CR33] Connor, L. T., Dunlosky, J., & Hertzog, C. (1997). Age-related differences in absolute but not relative metamemory accuracy. *Psychology and Aging,**12*, 50–71. 9100268 10.1037//0882-7974.12.1.50

[CR34] Craik, F. I. M., & Bosman, B. A. (1992). Age-related changes in memory and learning. In H. Bouma & J. A. M. Graafmans (Eds.), *Gerontechnology* (pp. 79–92). IOS Press.

[CR35] Craik, F. I. M., Govoni, R., Naveh-Benjamin, M., & Anderson, N. C. (1996). The effects of divided attention on encoding and retrieval processes in human memory. *Journal of Experimental Psychology: General,**125*, 159–180. 8683192 10.1037//0096-3445.125.2.159

[CR36] Doerksen, S., & Shimamura, A. P. (2001). Source memory enhancement for emotional words. *Emotion,**1*, 5–11. 12894807 10.1037/1528-3542.1.1.5

[CR37] Double, K. S., & Birney, D. P. (2019). Reactivity to measures of metacognition. *Frontiers in Psychology,**10*, 2755. 31866919 10.3389/fpsyg.2019.02755 PMC6908488

[CR38] Double, K. S., Birney, D. P., & Walker, S. A. (2018). A meta-analysis and systematic review of reactivity to judgements of learning. *Memory,**26*, 741–750. 29161973 10.1080/09658211.2017.1404111

[CR39] Dougherty, M. R., Scheck, P., Nelson, T. O., & Narens, L. (2005). Using the past to predict the future. *Memory & Cognition,**33*, 1096–1115. 16496729 10.3758/bf03193216

[CR40] Dror, I. E., & Harnad, S. (2008). Offloading cognition onto cognitive technology. In I. E. Dror & S. Harnad (Eds.), *Cognition distributed: How cognitive technology extends our minds* (pp. 1–23). John Benjamins.

[CR41] Dunlosky, J., & Hertzog, C. (1997). Older and younger adults use a functionally identical algorithm to select items for restudy during multitrial learning. *Journal of Gerontology: Psychological Sciences,**52B*, 178–186. 10.1093/geronb/52b.4.p178 9224442

[CR42] Dunlosky, J., & Matvey, G. (2001). Empirical analysis of the intrinsic-extrinsic distinction of judgments of learning (JOLs): Effects of relatedness and serial position on JOLs. *Journal of Experimental Psychology: Learning, Memory, and Cognition,**27*, 1180–1191. 11550746 10.1037//0278-7393.27.5.1180

[CR43] Dunlosky, J., & Ariel, R. (2011a). The influence of agenda-based and habitual processes on item selection during study. *Journal of Experimental Psychology: Learning, Memory, and Cognition,**37*, 899–912. 21480756 10.1037/a0023064

[CR44] Dunlosky, J., & Ariel, R. (2011b). Self-regulated learning and the allocation of study time. In B. H. Ross (Ed.), *The psychology of learning and motivation* (vol. 54, pp. 103–140). Academic Press.

[CR45] Dunlosky, J., Mueller, M. L., & Thiede, K. W. (2016). Methodology for investigating human metamemory: Problems and pitfalls. In J. Dunlosky & S. K. Tauber (Eds.), *Oxford library of psychology. The Oxford handbook of metamemory* (pp. 23–37). Oxford University Press.

[CR46] Ebner, N. C., Freund, A. M., & Baltes, P. B. (2006). Developmental changes in personal goal orientation from young to late adulthood: From striving for gains to maintenance and prevention of losses. *Psychology and Aging,**21*, 664–678. 17201488 10.1037/0882-7974.21.4.664

[CR47] Egner, T. (2017). *The Wiley handbook of cognitive control*. Wiley Blackwell.

[CR48] Elliott, B. L., & Brewer, G. A. (2019). Divided attention selectively impairs value-directed encoding. *Collabra Psychology,**5*, 4.

[CR49] Elliott, B. L., Blais, C., McClure, S. M., & Brewer, G. A. (2020a). Neural correlates underlying the effect of reward value on recognition memory. *NeuroImage,**206*, 116296. 31648002 10.1016/j.neuroimage.2019.116296 PMC8979913

[CR50] Elliott, B. L., McClure, S. M., & Brewer, G. A. (2020b). Individual differences in value-directed remembering. *Cognition,**201*, 104275. 32387721 10.1016/j.cognition.2020.104275 PMC8932348

[CR51] Elliott, B. L., D’Ardenne, K., Murty, V. P., Brewer, G. A., & McClure, S. M. (2022). Midbrain–hippocampus structural connectivity selectively predicts motivated memory encoding. *Journal of Neuroscience,**42*, 9426–9434. 36332978 10.1523/JNEUROSCI.0945-22.2022 PMC9794367

[CR52] Eskritt, M., & Ma, S. (2014). Intentional forgetting: Note-taking as a naturalistic example. *Memory & Cognition,**42*, 237–246. 24014168 10.3758/s13421-013-0362-1

[CR53] Fawcett, J. M., & Hulbert, J. C. (2020). The many faces of forgetting: Toward a constructive view of forgetting in everyday life. *Journal of Applied Research in Memory and Cognition,**9*, 1–18.

[CR54] Finn, B. (2008). Framing effects on metacognitive monitoring and control. *Memory & Cognition,**36*, 813–821. 18604963 10.3758/mc.36.4.813 PMC2582158

[CR55] Freund, A. M. (2008). Successful aging as management of resources: The role of selection, optimization, and compensation. *Research in Human Development,**5*, 94–106.

[CR56] Freund, A. M., & Baltes, P. B. (2000). The orchestration of selection, optimization and compensation: An action-theoretical conceptualization of a theory of developmental regulation. In W. J. Perrig & A. Grob (Eds.), *Control of human behavior, mental processes, and consciousness: Essays in honor of the 60th birthday of August Flammer* (pp. 35–58). Erlbaum.

[CR57] Friedman, M. C., McGillivray, S., Murayama, K., & Castel, A. D. (2015). Memory for medication side effects in younger and older adults: The role of subjective and objective importance. *Memory & Cognition,**43*, 206–215. 25331278 10.3758/s13421-014-0476-0 PMC4329267

[CR58] Gonzalez, R., & Nelson, T. O. (1996). Measuring ordinal association in measures that contain tied scores. *Psychological Bulletin,**119*, 159–165. 8559859 10.1037/0033-2909.119.1.159

[CR59] Griffin, M. L., Benjamin, A. S., Sahakyan, L., & Stanley, S. E. (2019). A matter of priorities: High working memory enables (slightly) superior value-directed remembering. *Journal of Memory and Language,**108*, 104032.

[CR60] Hanczakowski, M., Zawadzka, K., Pasek, T., & Higham, P. A. (2013). Calibration of metacognitive judgments: Insights from the underconfidence-with-practice effect. *Journal of Memory and Language,**69*, 429–444.

[CR61] Hargis, M. B., & Castel, A. D. (2017). Younger and older adults’ associative memory for social information: The role of information importance. *Psychology and Aging,**32*, 325–330. 28581330 10.1037/pag0000171 PMC5499679

[CR62] Hargis, M. B., & Castel, A. D. (2018a). Improving medication understanding and adherence using principles of memory and metacognition. *Policy Insights from Behavioral and Brain Sciences,**5*, 147–154. 10.1177/2372732218781643 PMC6758907 31552287

[CR63] Hargis, M. B., & Castel, A. D. (2018b). Younger and older adults’ associative memory for medication interactions of varying severity. *Memory,**26*, 1151–1158. 29463183 10.1080/09658211.2018.1441423 PMC6168289

[CR64] Hargis, M. B., & Castel, A. D. (2019). Knowing what others know: Younger and older adults’ perspective-taking and memory for medication information. *Journal of Applied Research in Memory and Cognition,**8*, 481–493. 34055581 10.1016/j.jarmac.2019.09.004 PMC8158662

[CR65] Henkel, L. A. (2014). Point-and-shoot memories: The influence of taking photos on memory for a museum tour. *Psychological Science,**25*, 396–402. 24311477 10.1177/0956797613504438

[CR66] Hennessee, J. P., Patterson, T. K., Castel, A. D., & Knowlton, B. J. (2019). Forget me not: Encoding processes in value-directed remembering. *Journal of Memory and Language,**106*, 29–39.

[CR67] Hertzog, C., & Dunlosky, J. (2011). Metacognition in later adulthood: Spared monitoring can benefit older adults’ self-regulation. *Current Directions in Psychological Science,**20*, 167–173. 24478539 10.1177/0963721411409026 PMC3903298

[CR68] Hess, T. M. (2005). Memory and aging in context. *Psychological Bulletin,**131*, 383–406. 15869334 10.1037/0033-2909.131.3.383

[CR69] Higham, P. A., Zawadzka, K., & Hanczakowski, M. (2016). Internal mapping and its impact on measures of absolute and relative metacognitive accuracy. In J. Dunlosky & S. K. Tauber (Eds.), *The Oxford handbook of metamemory* (pp. 65–80). Oxford University Press.

[CR70] Hoover, K. M., Murphy, D. H., Middlebrooks, C. D., & Castel, A. D. (2024). The effect of time constraints on value-directed long-term memory in younger and older adults. *Psychology and Aging,**39*, 166–179. 38271074 10.1037/pag0000795 PMC10932845

[CR71] Janes, J. L., Rivers, M. L., & Dunlosky, J. (2018). The influence of making judgments of learning on memory performance: Positive, negative, or both? *Psychonomic Bulletin & Review,**25*, 2356–2364. 29611141 10.3758/s13423-018-1463-4

[CR72] Kelly, M. O., & Risko, E. F. (2019a). The isolation effect when offloading memory. *Journal of Applied Research in Memory and Cognition,**8*, 471–480.

[CR73] Kelly, M. O., & Risko, E. F. (2019b). Offloading memory: Serial position effects. *Psychonomic Bulletin & Review,**26*, 1347–1353. 31161530 10.3758/s13423-019-01615-8

[CR74] Kensinger, E. A., & Corkin, S. (2003). Memory enhancement for emotional words: Are emotional words more vividly remembered than neutral words? *Memory & Cognition,**31*, 1169–1180. 15058678 10.3758/bf03195800

[CR75] Kleinsmith, L. J., & Kaplan, S. (1963). Paired-associate learning as a function of arousal and interpolated interval. *Journal of Experimental Psychology,**65*, 190–193. 14033436 10.1037/h0040288

[CR76] Knowlton, B. J., & Castel, A. D. (2022). Memory and reward-based learning: A value-directed remembering perspective. *Annual Review of Psychology,**73*, 25–52. 34587778 10.1146/annurev-psych-032921-050951

[CR77] Koriat, A. (1997). Monitoring one’s own knowledge during study: A cue-utilization approach to judgments of learning. *Journal of Experimental Psychology: General,**126*, 349–370.

[CR78] Koriat, A. (2015). Knowing by doing: When metacognitive monitoring follows metacognitive control. In D. S. Lindsay, C. M. Kelley, A. P. Yonelinas, & H. L. Roediger II. (Eds.), *Psychology Press festschrift series. Remembering: Attributions, processes, and control in human memory: Essays in honor of Larry Jacoby* (pp. 185–197). Psychology Press.

[CR79] Koriat, A., Bjork, R. A., Sheffer, L., & Bar, S. K. (2004). Predicting one’s own forgetting: The role of experience-based and theory-based processes. *Journal of Experimental Psychology: General,**133*, 643–656. 15584811 10.1037/0096-3445.133.4.643

[CR80] Koriat, A., Ma’ayan, H., & Nussinson, R. (2006). The intricate relationships between monitoring and control in metacognition: Lessons for the cause-and-effect relation between subjective experience and behavior. *Journal of Experimental Psychology: General,**135*, 36–69. 16478315 10.1037/0096-3445.135.1.36

[CR81] Kornell, N., & Metcalfe, J. (2006). Study efficacy and the region of proximal learning framework. *Journal of Experimental Psychology: Learning, Memory, and Cognition,**32*, 609–622. 16719670 10.1037/0278-7393.32.3.609

[CR82] Kornell, N., Rhodes, M. G., Castel, A. D., & Tauber, S. K. (2011). The ease of processing heuristic and the stability bias: Dissociating memory, memory beliefs, and memory judgments. *Psychological Science,**22*, 787–794. 21551341 10.1177/0956797611407929

[CR83] LaBar, K. S., & Phelps, E. A. (1998). Arousal-mediated memory consolidation: Role of the medial temporal lobe in humans. *Psychological Science,**9*, 490–493.

[CR84] Leonesio, R. J., & Nelson, T. O. (1990). Do different metamemory judgments tap the same underlying aspects of memory? *Journal of Experimental Psychology: Learning, Memory, and Cognition,**16*, 464–470. 2140404 10.1037//0278-7393.16.3.464

[CR85] Lu, X., Kelly, M. O., & Risko, E. F. (2020). Offloading information to an external store increases false recall. *Cognition,**205*, 104428. 32863020 10.1016/j.cognition.2020.104428

[CR86] Luna, K., Martín-Luengo, B., & Albuquerque, P. B. (2018). Do delayed judgements of learning reduce metamemory illusions? A meta-analysis. *Quarterly Journal of Experimental Psychology,**71*, 1626–1636. 10.1080/17470218.2017.1343362 28856962

[CR87] Luna, K., Nogueira, M., & Albuquerque, P. B. (2019). Words in larger font are perceived as more important: explaining the belief that font size affects memory. *Memory,**27*, 555–560. 30293477 10.1080/09658211.2018.1529797

[CR88] MacLeod, C. (1998). Directed forgetting. In J. Golding & C. M. MacLeod (Eds.), *Intentional forgetting: Interdisciplinary approaches* (pp. 139–172). Erlbaum.

[CR89] Madan, C. R. (2017). Motivated cognition: Effects of reward, emotion, and other motivational factors across a variety of cognitive domains. *Collabra: Psychology,**3*, 24.

[CR90] Marsh, E. J., & Rajaram, S. (2019). The digital expansion of the mind: Implications of internet usage for memory and cognition. *Journal of Applied Research in Memory and Cognition,**8*, 1–14.

[CR91] Masson, M. E. J., & Rotello, C. M. (2009). Sources of bias in the Goodman-Kruskal gamma coefficient measure of association: Implications for studies of metacognitive processes. *Journal of Experimental Psychology: Learning, Memory, and Cognition,**35*, 509–527. 19271863 10.1037/a0014876

[CR92] Mata, R., Schooler, L. J., & Rieskamp, J. (2007). The aging decision maker: cognitive aging and the adaptive selection of decision strategies. *Psychology and Aging,**22*, 796–810. 18179298 10.1037/0882-7974.22.4.796

[CR93] McCabe, D. P., & Soderstrom, N. C. (2011). Recollection-based metamemory judgments are more accurate than those based on confidence: Judgments of remembering and knowing (JORKs). *Journal of Experimental Psychology: General,**140*, 605–621. 21707208 10.1037/a0024014

[CR94] McGillivray, S., & Castel, A. D. (2011). Betting on memory leads to metacognitive improvement in younger and older adults. *Psychology and Aging,**26*, 137–142. 21417541 10.1037/a0022681

[CR95] McGillivray, S., & Castel, A. D. (2017). Older and younger adults’ strategic control of metacognitive monitoring: The role of consequences, task experience and prior knowledge. *Experimental Aging Research,**43*, 362–374. 10.1080/0361073X.2017.1298956 PMC6756177 28358293

[CR96] Metcalfe, J., & Kornell, N. (2005). A region of proximal learning model of study time allocation. *Journal of Memory and Language,**52*, 463–477.

[CR97] Metcalfe, J., & Finn, B. (2008). Evidence that judgments of learning are causally related to study choice. *Psychonomic Bulletin & Review,**15*, 174–179. 18605499 10.3758/pbr.15.1.174

[CR98] Middlebrooks, C. D., McGillivray, S., Murayama, K., & Castel, A. D. (2016a). Memory for allergies and health foods: How younger and older adults strategically remember critical health information. *Journal of Gerontology: Psychological Sciences,**71*, 389–399. 10.1093/geronb/gbv032 PMC5013922 25975293

[CR99] Middlebrooks, C. D., Murayama, K., & Castel, A. D. (2016b). The value in rushing: Memory and selectivity when short on time. *Acta Psychologica,**170*, 1–9. 27305652 10.1016/j.actpsy.2016.06.001 PMC5045783

[CR100] Middlebrooks, C. D., Kerr, T. K., & Castel, A. D. (2017). Selectively distracted: Divided attention and memory for important information. *Psychological Science,**28*, 1103–1115. 28604267 10.1177/0956797617702502 PMC5546942

[CR101] Mitchum, A. L., Kelley, C. M., & Fox, M. C. (2016). When asking the question changes the ultimate answer: Metamemory judgments change memory. *Journal of Experimental Psychology: General,**145*, 200–219. 27045282 10.1037/a0039923

[CR102] Mueller, M. L., & Dunlosky, J. (2016). How beliefs can impact judgments of learning: Evaluating analytic processing theory with beliefs about fluency. *Journal of Memory and Language,**93*, 245–258.

[CR103] Mueller, M. L., Dunlosky, J., Tauber, S. K., & Rhodes, M. G. (2014). The font-size effect on judgments of learning: Does it exemplify fluency effects of reflect people’s beliefs about memory? *Journal of Memory and Language,**70*, 1–12.

[CR104] Murayama, K., Sakaki, M., Yan, V. X., & Smith, G. (2014). Type-1 error inflation in the traditional by-participant analysis to metamemory accuracy: A generalized mixed effects model perspective. *Journal of Experimental Psychology: Learning, Memory & Cognition,**40*, 1287–1306. 24911135 10.1037/a0036914

[CR105] Murphy, D. H. (2023a). Does value structure influence measures of memory selectivity? *Memory,**31*, 1074–1088. 37279188 10.1080/09658211.2023.2221006

[CR106] Murphy, D. H. (2023b). Strategic offloading: How the value of to-be-remembered information influences offloading decision-making. *Applied Cognitive Psychology,**37*, 749–767.

[CR107] Murphy, N. A., & Isaacowitz, D. M. (2008). Preferences for emotional information in older and younger adults: A meta-analysis of memory and attention tasks. *Psychology and Aging,**23*, 263–286. 18573002 10.1037/0882-7974.23.2.263

[CR108] Murphy, D. H., & Castel, A. D. (2020). Responsible remembering: How metacognition impacts adaptive selective memory. *Zeitschrift für Psychologie,**228*, 301–303.

[CR109] Murphy, D. H., & Castel, A. D. (2021a). Metamemory that matters: Judgments of importance can engage responsible remembering. *Memory,**29*, 271–283. 33726614 10.1080/09658211.2021.1887895 PMC8009862

[CR110] Murphy, D. H., & Castel, A. D. (2021b). Responsible remembering and forgetting as contributors to memory for important information. *Memory & Cognition,**49*, 895–911. 33474691 10.3758/s13421-021-01139-4 PMC8238741

[CR111] Murphy, D. H., & Castel, A. D. (2021c). The role of attention and aging in the retrieval dynamics of value-directed remembering. *Quarterly Journal of Experimental Psychology,**75*, 954–968. 10.1177/17470218211046612 34467795

[CR112] Murphy, D. H., & Castel, A. D. (2022a). Differential effects of proactive and retroactive interference in value-directed remembering for younger and older adults. *Psychology and Aging,**37*, 787–799. 36048043 10.1037/pag0000707 PMC10029347

[CR113] Murphy, D. H., & Castel, A. D. (2022b). Responsible remembering and forgetting in younger and older adults. *Experimental Aging Research,**48*, 455–473. 35142260 10.1080/0361073X.2022.2033592 PMC9363524

[CR114] Murphy, D. H., & Castel, A. D. (2022c). Selective remembering and directed forgetting are influenced by similar stimulus properties. *Memory,**30*, 1130–1147. 35730700 10.1080/09658211.2022.2092152

[CR115] Murphy, D. H., & Knowlton, B. J. (2022d). Framing effects in value-directed remembering. *Memory & Cognition,**50*, 1350–1361. 35488098 10.3758/s13421-022-01317-y PMC9365741

[CR116] Murphy, D. H., & Castel, A. D. (2023a). Age-related differences in memory when offloading important information. *Psychology and Aging,**38*, 415–427. 37166862 10.1037/pag0000750 PMC10524137

[CR117] Murphy, D. H., & Castel, A. D. (2023b). Age-related differences in overcoming interference when selectively remembering important information. *Experimental Aging Research,**50*, 190–205. 36744521 10.1080/0361073X.2023.2176629 PMC10404302

[CR118] Murphy, D. H., & Castel, A. D. (2023c). Responsible attention: The effect of divided attention on metacognition and responsible remembering. *Psychological Research,**87*, 1085–1100. 35838835 10.1007/s00426-022-01711-w PMC10191991

[CR119] Murphy, D. H., & Castel, A. D. (2024). Serial and strategic processing in younger and older adults. *Aging, Neuropsychology and Cognition*. 10.1080/13825585.2024.2371177 Advance online publication 10.1080/13825585.2024.2371177 PMC11664019 38909315

[CR120] Murphy, D. H., Agadzhanyan, K., Whatley, M. C., & Castel, A. D. (2021). Metacognition and fluid intelligence in value-directed remembering. *Metacognition and Learning,**16*, 685–709.

[CR121] Murphy, D. H., Huckins, S. C., Rhodes, M. G., & Castel, A. D. (2022a). The effect of perceptual processing fluency and value on metacognition and remembering. *Psychonomic Bulletin & Review,**29*, 910–921. 34846689 10.3758/s13423-021-02030-8

[CR122] Murphy, D. H., Schwartz, S. T., & Castel, A. D. (2022b). Serial and strategic memory processes in goal-directed selective remembering. *Cognition,**225*, 105178. 35644091 10.1016/j.cognition.2022.105178

[CR123] Murphy, D. H., Halamish, V., Rhodes, M. G., & Castel, A. D. (2023a). How evaluating memorability can lead to unintended consequences. *Metacognition and Learning,**18*, 375–403.

[CR124] Murphy, D. H., Hargis, M. B., & Castel, A. D. (2023b). Younger and older adults’ strategic use of associative memory and metacognitive control when learning foreign vocabulary words of varying importance. *Psychology and Aging,**38*, 103–116. 36757965 10.1037/pag0000730 PMC10038181

[CR125] Murphy, D. H., Hoover, K. M., & Castel, A. D. (2023c). Strategic metacognition: Self-paced study time and responsible remembering. *Memory & Cognition,**51*, 234–251. 35349110 10.3758/s13421-022-01307-0

[CR126] Murphy, D. H., Castel, A. D., & Knowlton, B. J. (2024a). Age-related similarities and differences in framing selective memory in terms of gains and losses. *Experimental Aging Research,**50*, 506–521. 37409470 10.1080/0361073X.2023.2233366 PMC10770296

[CR127] Murphy, D. H., Hoover, K. M., & Castel, A. D. (2024b). Age-related differences in selective associative memory: Implications for responsible remembering. *Aging, Neuropsychology and Cognition,**31*(4), 682–704. 10.1080/13825585.2023.2249189 37594007 10.1080/13825585.2023.2249189 PMC10874462

[CR128] Murphy, D. H., Hoover, K. M., Knowlton, B. J., & Castel, A. D. (2024c). Memory and strategic value-directed remembering in younger and older adults. *Psychology and Aging,**39*(2), 166–179. Manuscript under review. 38271074 10.1037/pag0000795 PMC10932845

[CR129] Murphy, D. H., Schwartz, S. T., & Castel, A. D. (2024d). Value-directed retrieval: The effects of divided attention at encoding and retrieval on memory selectivity and retrieval dynamics. *Journal of Experimental Psychology: Learning, Memory, and Cognition,**50*, 17–38. 37326541 10.1037/xlm0001264

[CR130] Murphy, D. H., Rhodes, M. G., & Castel, A. D. (2024e). The perceived importance of words in large font guides learning and selective memory. *Memory & Cognition*. 10.3758/s13421-024-01555-2 PMC11522127 38641757

[CR131] Murphy, D. H., Hoover, K. M., Castel, A. D., & Knowlton, B. J. (in press). Memory and automatic processing of valuable information in younger and older adults. *Aging, Neuropsychology and Cognition*. 10.1080/13825585.2024.2360226 PMC11604819 38809169

[CR132] Nairne, J. S. (2010). Adaptive memory: Evolutionary constraints on remembering. In B. H. Ross (Ed.), *The psychology of learning and motivation* (vol. 53, pp. 1–32). Academic Press.

[CR133] Nairne, J. S. (2013). Adaptive memory: Controversies and future directions. In B. L. Schwartz, M. L. Howe, M. P. Toglia, & H. Otgaar (Eds.), *What is adaptive about adaptive memory?* (pp. 308–321). Oxford University Press.

[CR134] Nairne, J. S. (2015). Adaptive memory: Novel findings acquired through forward engineering. In D. S. Lindsay, C. M. Kelley, A. P. Yonelinas, & H. L. Roediger (Eds.), *Remembering: Attributions, processes, and control in human memory. *Psychology Press.

[CR135] Nairne, J. S., & Pandeirada, J. N. S. (2008). Adaptive memory: Remembering with a stone-age brain. *Current Directions in Psychological Science,**17*, 239–243.

[CR136] Nairne, J. S., & Pandeirada, J. N. S. (2010). Adaptive memory: Ancestral priorities and the mnemonic value of survival processing. *Cognitive Psychology,**61*, 1–22. 20206924 10.1016/j.cogpsych.2010.01.005

[CR137] Nairne, J. S., Thompson, S. R., & Pandeirada, J. N. S. (2007). Adaptive memory: Survival processing enhances retention. *Journal of Experimental Psychology: Learning, Memory, and Cognition,**33*, 263–273. 17352610 10.1037/0278-7393.33.2.263

[CR138] Naveh-Benjamin, M. (2000). Adult age differences in memory performance: Tests of an associative deficit hypothesis. *Journal of Experimental Psychology: Learning, Memory, and Cognition,**26*, 1170–1187. 11009251 10.1037//0278-7393.26.5.1170

[CR139] Nelson, T. O. (1984). A comparison of current measures of the accuracy of feeling-of-knowing predictions. *Psychological Bulletin,**84*, 93–116. 6544431

[CR140] Nelson, T. O. (1996). Consciousness and metacognition. *American Psychologist,**51*, 102–116.

[CR141] Nelson, T. O., & Narens, L. (1990). Metamemory: A theoretical framework and some new findings. In G. H. Bower (Ed.), *The psychology of learning and motivation* (80th ed., pp. 125–173). Academic Press.

[CR142] Nelson, T. O., Dunlosky, J., Graf, A., & Narens, L. (1994). Utilization of metacognitive judgments in the allocation of study during multitrial learning. *Psychological Science,**5*, 207–213.

[CR143] Park, D. C., & Festini, S. B. (2017). Theories of memory and aging: A look at the past and a glimpse of the future. *Journals of Gerontology: Psychological Sciences,**72*, 82–90. 10.1093/geronb/gbw066 PMC5156492 27257229

[CR144] Park, J. S., Kelly, M. O., Hargis, M. B., & Risko, E. F. (2022). The effect of external store reliance on actual and predicted value-directed remembering. *Psychonomic Bulletin & Review,**29*, 1367–1376. 35182387 10.3758/s13423-022-02064-6

[CR145] Peng, Y., & Tullis, J. G. (2021). Dividing attention impairs metacognitive control more than monitoring. *Psychonomic Bulletin & Review,**28*, 2065–2075. 10.3758/s13423-021-01950-9 PMC8205317 34131889

[CR146] Queen, T. L., Hess, T. M., Ennis, G. E., Dowd, K., & Grühn, D. (2012). Information search and decision making: Effects of age and complexity on strategy use. *Psychology and Aging,**27*, 817–824. 22663157 10.1037/a0028744 PMC3435436

[CR147] Rhodes, M. G. (2016). Judgments of learning. In J. Dunlosky & S. K. Tauber (Eds.), *The Oxford handbook of metamemory* (pp. 65–80). Oxford University Press.

[CR148] Rhodes, M. G., & Castel, A. D. (2008). Memory predictions are influenced by perceptual information: Evidence for metacognitive illusions. *Journal of Experimental Psychology: General,**137*, 615–625. 18999356 10.1037/a0013684

[CR149] Rhodes, M. G., & Castel, A. D. (2009). Metacognitive illusions for auditory information: Effects on monitoring and control. *Psychonomic Bulletin & Review,**16*, 550–554. 19451383 10.3758/PBR.16.3.550

[CR150] Rhodes, M. G., & Tauber, S. K. (2011). The influence of delaying judgments of learning on metacognitive accuracy: A meta-analytic review. *Psychological Bulletin,**137*, 131–148. 21219059 10.1037/a0021705

[CR151] Risko, E. F., & Dunn, T. L. (2015). Storing information in-the-world: Metacognition and cognitive offloading in a short-term memory task. *Consciousness and Cognition,**36*, 61–74. 26092219 10.1016/j.concog.2015.05.014

[CR152] Risko, E. F., & Gilbert, S. J. (2016). Cognitive offloading. *Trends in Cognitive Sciences,**20*, 676–688. 27542527 10.1016/j.tics.2016.07.002

[CR153] Robison, M. K., & Unsworth, N. (2017). Working memory capacity, strategic allocation of study time, and value-directed remembering. *Journal of Memory and Language,**93*, 231–244.

[CR154] Roediger, H. L., III. (1974). Inhibiting effects of recall. *Memory & Cognition,**2*, 261–269. 24214752 10.3758/BF03208993

[CR155] Roediger, H. L., III., & Schmidt, S. R. (1980). Output interference in the recall of categorized and paired associate lists. *Journal of Experimental Psychology: Human Learning & Memory,**6*, 91–105.

[CR156] Rubin, D. C., & Friendly, M. (1986). Predicting which words get recalled: Measures of free recall, availability, goodness, emotionality, and pronunciability for 925 nouns. *Memory & Cognition,**14*, 79–94. 3713510 10.3758/bf03209231

[CR157] Salthouse, T. A. (2010). Selective review of cognitive aging. *Journal of the International Neuropsychological Society,**16*, 754–760. 20673381 10.1017/S1355617710000706 PMC3637655

[CR158] Schwartz, B. L., & Efklides, A. (2012). Metamemory and memory efficiency: Implications for student learning. *Journal of Applied Research in Memory and Cognition,**1*, 145–151.

[CR159] Serra, M. J., & England, B. D. (2012). Magnitude and accuracy differences between judgments of remembering and forgetting. *Quarterly Journal of Experimental Psychology,**65*, 2231–2257. 10.1080/17470218.2012.685081 22630784

[CR160] Shigemune, Y., Tsukiura, T., Kambara, T., & Kawashima, R. (2014). Remembering with gains and losses: Effects of monetary reward and punishment on successful encoding activation of source memories. *Cerebral Cortex,**24*, 1319–1331. 23314939 10.1093/cercor/bhs415 PMC3977621

[CR161] Siegel, A. L. M., & Castel, A. D. (2018). Memory for important item-location associations in younger and older adults. *Psychology and Aging,**33*, 30–45. 29494176 10.1037/pag0000209 PMC5836789

[CR162] Siegel, A. L. M., Schwartz, S. T., & Castel, A. D. (2021). Selective memory disrupted in intra-modal dual-task encoding conditions. *Memory & Cognition,**49*(7), 1453–1472. 33763815 10.3758/s13421-021-01166-1 PMC8460703

[CR163] Smith, A. D. (1971). Output interference and organized recall from long-term memory. *Journal of Verbal Learning and Verbal Behavior,**10*, 400–408.

[CR164] Smith, A. D. (1974). Response interference with organized recall in the aged. *Developmental Psychology,**10*, 867–870.

[CR165] Soderstrom, N. C., & McCabe, D. P. (2011). The interplay between value and relatedness as bases for metacognitive monitoring and control: Evidence for agenda-based monitoring. *Journal of Experimental Psychology: Learning, Memory, and Cognition,**37*, 1236–1242. 21574750 10.1037/a0023548

[CR166] Soderstrom, N. C., Clark, C. T., Halamish, V., & Bjork, E. L. (2015). Judgments of learning as memory modifiers. *Journal of Experimental Psychology: Learning, Memory, and Cognition,**41*, 553–558. 25528101 10.1037/a0038388

[CR167] Son, L. K., & Metcalfe, J. (2000). Metacognitive and control strategies in study-time allocation. *Journal of Experimental Psychology: Learning, Memory, and Cognition,**26*, 204–221. 10682298 10.1037//0278-7393.26.1.204

[CR168] Sparrow, B., Liu, J., & Wegner, D. M. (2011). Google effects on memory: Cognitive consequences of having information at our fingertips. *Science,**333*, 776–778. 21764755 10.1126/science.1207745

[CR169] Spellman, B. A., & Bjork, R. A. (1992). When predictions create reality: Judgments of learning may alter what they are intended to assess. *Psychological Science,**5*, 315–316.

[CR170] Stefanidi, A., Ellis, D. M., & Brewer, G. A. (2018). Free recall dynamics in value-directed remembering. *Journal of Memory and Language,**100*, 18–31.

[CR171] Storm, B. C. (2011). The benefit of forgetting in thinking and remembering. *Current Directions in Psychological Science,**20*, 291–295.

[CR172] Storm, B. C., & Stone, S. M. (2014). Saving-enhanced memory: The benefits of saving on the learning and remembering of new information. *Psychological Science,**26*, 182–188. 25491269 10.1177/0956797614559285

[CR173] Tauber, S. K., & Rhodes, M. G. (2012). Measuring memory monitoring with judgments of retention interval (JOR). *Quarterly Journal of Experimental Psychology,**65*, 1376–1396. 10.1080/17470218.2012.656665 22524495

[CR174] Thiede, K. W., & Dunlosky, J. (1999). Toward a general model of self-paced study: An analysis of selection of items for study and self-paced study time. *Journal of Experimental Psychology: Learning, Memory, and Cognition,**25*, 1024–1037.

[CR175] Tiede, H. L., & Leboe, J. P. (2009). Metamemory judgments and the benefits of repeated study: Improving recall predictions through the activation of appropriate knowledge. *Journal of Experimental Psychology: Learning, Memory, and Cognition,**35*, 822–828. 19379052 10.1037/a0015122

[CR176] Underwood, B. J. (1975). Individual differences as a crucible in theory construction. *American Psychologist,**30*, 128–134.

[CR177] Undorf, M., & Bröder, A. (2019). Cue integration in metamemory judgments is strategic. *Quarterly Journal of Experimental Psychology,**73*(4), 629–642. 10.1177/1747021819882308 31561744

[CR178] Undorf, M., & Zimdahl, M. F. (2019). Metamemory and memory for a wide range of font sizes: What is the contribution of perceptual fluency? *Journal of Experimental Psychology: Learning, Memory, and Cognition,**45*, 97–109. 29698050 10.1037/xlm0000571

[CR179] Vuorre, M., & Bolger, N. (2018). Within-subject mediation analysis for experimental data in cognitive psychology and neuroscience. *Behavior Research Methods,**50*, 2125–2143. 29247385 10.3758/s13428-017-0980-9

[CR180] Whatley, M. C., Murphy, D. H., Silaj, K. M., & Castel, A. D. (2021). Motivated memory for what matters most: How older adults (selectively) focus on important information and events using schematic support, metacognition, and meaningful goals. In G. Sedek, T. M. Hess, & D. R. Touron (Eds.), *Multiple pathways of cognitive aging: Motivational and contextual influences. *Oxford University Press.

